# Role of pyruvate in maintaining cell viability and energy production under high-glucose conditions

**DOI:** 10.1038/s41598-021-98082-w

**Published:** 2021-09-23

**Authors:** Hideji Yako, Naoko Niimi, Ayako Kato, Shizuka Takaku, Yasuaki Tatsumi, Yasumasa Nishito, Koichi Kato, Kazunori Sango

**Affiliations:** 1 Diabetic Neuropathy Project, Tokyo Metropolitan Institute of Medical Science, 2-1-6 Kamikitazawa, Setagaya-ku, Tokyo, 156-8506 Japan; 2 Laboratory of Medicine, Aichi Gakuin University, School of Pharmacy, Nagoya, Japan; 3 Basic Technology Research Center, Tokyo Metropolitan Institute of Medical Science, Tokyo, Japan

**Keywords:** Metabolomics, Cell death, Mechanisms of disease

## Abstract

Pyruvate functions as a key molecule in energy production and as an antioxidant. The efficacy of pyruvate supplementation in diabetic retinopathy and nephropathy has been shown in animal models; however, its significance in the functional maintenance of neurons and Schwann cells under diabetic conditions remains unknown. We observed rapid and extensive cell death under high-glucose (> 10 mM) and pyruvate-starved conditions. Exposure of Schwann cells to these conditions led to a significant decrease in glycolytic flux, mitochondrial respiration and ATP production, accompanied by enhanced collateral glycolysis pathways (e.g., polyol pathway). Cell death could be prevented by supplementation with 2-oxoglutarate (a TCA cycle intermediate), benfotiamine (the vitamin B1 derivative that suppresses the collateral pathways), or the poly (ADP-ribose) polymerase (PARP) inhibitor, rucaparib. Our findings suggest that exogenous pyruvate plays a pivotal role in maintaining glycolysis–TCA cycle flux and ATP production under high-glucose conditions by suppressing PARP activity.

## Introduction

Peripheral neuropathy, as well as retinopathy and nephropathy, is a major complication of diabetes mellitus. While its pathogenesis remains unclear, continuous hyperglycemic insults diminish the microvascular supply in the peripheral nervous system (PNS) and induce various metabolic disorders [e.g. augmentation of the collateral glucose-utilizing pathways, formation of advanced glycation end-products (AGEs) and reactive oxygen species (ROS), endoplasmic reticulum stress, and reduced synthesis and/or availability of neurotrophic factors] in the PNS constituents, particularly neurons and Schwann cells^1^.

Endogenous pyruvate produced from glycose via glycolysis is a key molecule for energy production under aerobic and anaerobic conditions, whereas exogenous pyruvate is incorporated into cells via monocarboxylate transporters (MCTs)^2^ and predominantly functions as an antioxidant^3^.

Treatment with exogenous pyruvate has been shown to ameliorate hyperglycemia^4^, retinopathy^5^and nephropathy^6^ in streptozotocin-induced diabetic animals. In contrast, targeted gene disruption of murine pyruvate kinase, which catalyzes the conversion of phosphoenolpyruvate (PEP) to pyruvate, was shown to exacerbate diabetic nephropathy^7^. These findings suggest that both endogenous and exogenous pyruvate prevent and ameliorate diabetes and its complications. Serum pyruvate levels were lower in streptozotocin-induced diabetic rats with cognitive dysfunction than those in age-matched control rats^8^. Metabolic flux analysis using isotope-labelled glucose and pyruvate revealed significantly reduced amounts of citrate derived from extracellular glucose and increased amounts of citrate and malate derived from extracellular pyruvate in the sciatic nerves of diabetic *db/db* mice compared with control (*db*/+) littermates^9^. Furthermore, significant depletion of glycolytic and tricarboxylic acid (TCA) cycle intermediates in the peripheral nerves of diabetic mice was previously reported^9,10^. These findings led us to speculate that exogenous pyruvate could restore the impaired glycolysis–TCA cycle flux in the PNS under diabetic conditions. However, the efficacy of exogenous pyruvate as an antioxidant or glucose metabolism promoter at the cellular level remains unclear.

The present study examined the role of exogenous pyruvate in the functional maintenance of neurons and Schwann cells under high-glucose conditions. Primary cultured adult rat dorsal root ganglion (DRG) neurons and immortalized adult mouse Schwann (IMS32) cells were used, because these cells are recognized as useful tools for studying diabetic neuropathy^11^. Surprisingly, rapid and extensive death of both cell types was observed following exposure to high-glucose conditions in the absence of exogenous pyruvate. Cell death was attributed to impaired glycolytic flux with reduced glyceraldehyde 3-phosphate dehydrogenase (GAPDH) activity and disrupted mitochondrial respiration and ATP production. These findings highlight the pivotal role of exogenous pyruvate in maintaining glycolysis–TCA cycle flux under diabetic conditions.

## Results

### Pyruvate starvation under high-glucose conditions induced rapid and extensive cell death

**Figure 1:**
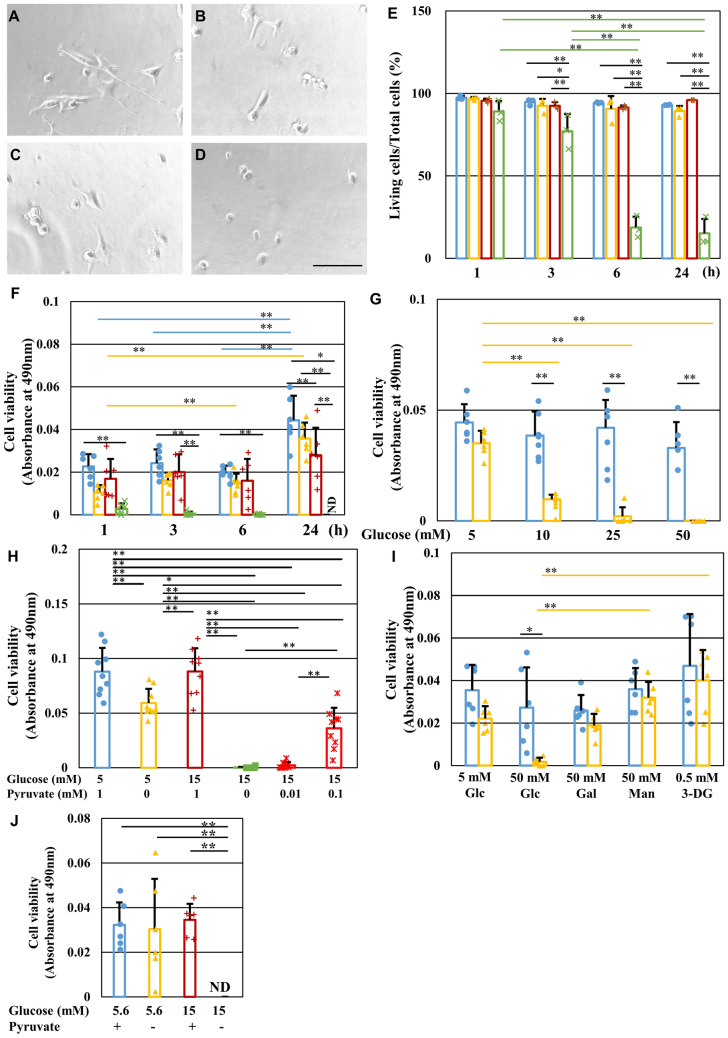
Pyruvate starvation induces rapid IMS32 Schwann cell death under high-glucose conditions. (**A–D**) Representative phase-contrast micrographs of IMS32 cells at 24 h in the [Glc 5 mM/Pyr ( +)] (**A**), [Glc 5 mM/Pyr (−)] (**B**), [Glc 50 mM/Pyr (+)] (**C**), and [Glc 50 mM/Pyr (−)] (**D**) groups. Scale bar represents 100 μm. (**E** and **F**) IMS32 cell viability at 1, 3, 6, and 24 h in the [Glc 5 mM/Pyr (+)] (blue), [Glc 5 mM/Pyr (−)] (yellow), [Glc 50 mM/Pyr (+)] (brown), and [Glc 50 mM/Pyr (−)] (green) groups was determined by Trypan blue staining (**E**) and MTS assay (**F**). (**G** and **I**) Cell viability at 24 h after exposure to 5, 10, 25, or 50 mM glucose (**G**) or 50 mM galactose, 50 mM mannitol or 0.5 mM 3-deoxyglucosone (**I**) in the presence (blue) or absence (yellow) of pyruvate was determined by MTS assay. (**H**) Cell viability at 24 h after exposure to [Glc 5 mM/Pyr 1 mM] (blue), [Glc 5 mM/Pyr (−)] (yellow), [Glc 15 mM/Pyr 1 mM] (brown), [Glc 15 mM/Pyr (−)] (green), and [Glc 15 mM/ Pyr 0.01 or 0.1 mM] (red) groups was determined by MTS assay. (**J**) Cell viability at 6 h after exposure to [Glc 5 mM/Pyr (+)] (blue), [Glc 5 mM/Pyr (−)] (yellow), [Glc 15 mM/Pyr (+)] (brown), and [Glc 15 mM/Pyr (−)] (green) in Tyrode’s solution with N2 supplement was determined. Values represent mean + SD from three (**E**), six (**F, G, I, J**) and nine (**H**) experiments (individual values are depicted as circles, triangles, pluses and crosses). * *P* < 0.05, ** *P* < 0.01. Glc, glucose; Gal, galactose; Man. Mannitol; 3-DG, 3-deoxyglucosone.

**Figure 2:**
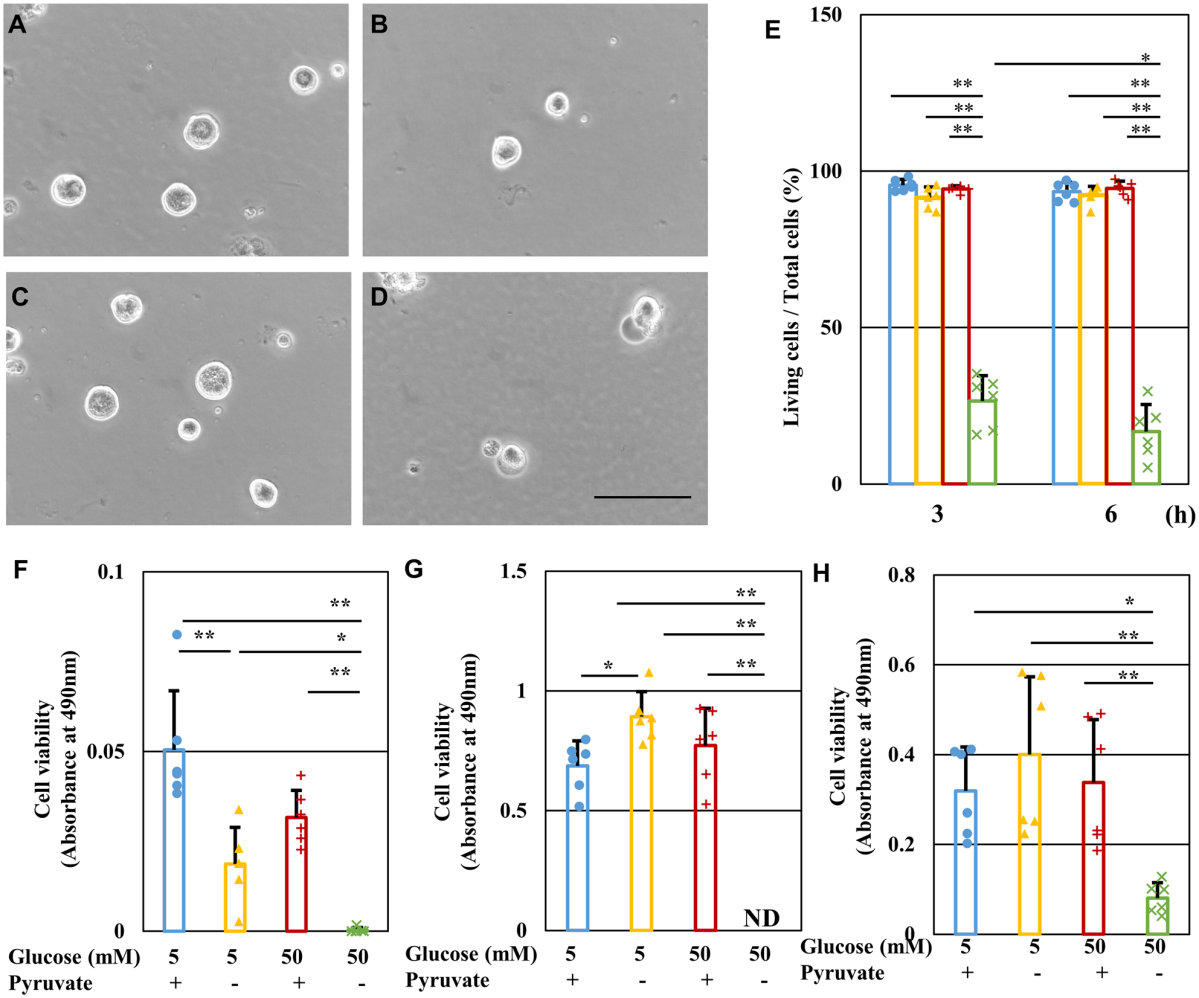
Pyruvate starvation induces rapid cell death of primary cultured rat DRG neurons, NSC-34 cells, MES13 cells and HAEC cells under high-glucose conditions. (**A-D**) Representative phase-contrast micrographs of DRG neurons at 24 h [Glc 5 mM/Pyr (+)] (**A**), [Glc 5 mM/Pyr (−)] (**B**), [Glc 50 mM/Pyr ( +)] (**C**), and [Glc 50 mM/Pyr (−)] (**D**) groups. Scale bar represents 100 μm. (**E**) The viability of DRG neurons at 3 and 6 h in the [Glc 5 mM/Pyr (+)] (blue), [Glc 5 mM/Pyr (−)] (yellow), [Glc 50 mM/Pyr (+)] (brown), and [Glc 50 mM/Pyr (−)] (green) groups was determined by Trypan blue staining. (**F–H**) The viability of NSC-34 cells (**F**), MES13 cells (**G**) and HAEC cells (**H**) at 24 h after exposure to the 4 conditions described above was determined by MTS assay. Values represent mean + SD from three (**E**) and six (**F–H**) experiments (individual values are depicted as circles, triangles, pluses and crosses). * *P* < 0.05, ** *P* < 0.01.

IMS32 Schwann cells were divided into four groups and maintained in media (DMEM with 5% FBS) containing normal (5 mM) or high (50 mM) glucose in the presence or absence of 1 mM pyruvate, termed the [Glc 5 mM/Pyr ( +)], [Glc 5 mM/Pyr (−)], [Glc 50 mM/Pyr ( +)] and [Glc 50 mM/Pyr (−)] groups, respectively (Fig. 1A–D). After 24 h, observation of the cells under a phase-contrast microscope revealed that IMS32 cells in the [Glc 5 mM/Pyr ( +)], [Glc 5 mM/Pyr (−)], and [Glc 50 mM/Pyr ( +)] groups (Fig. 1A–C) maintained a characteristic spindle-shaped morphology, whereas almost all the cells in the [Glc 50 mM/Pyr (−)] group (Fig. 1D) became round and shrunk. Consistent with these morphological features, a cell viability assay using Trypan blue stain revealed a steep decline in the mean viability ratios over time in the [Glc 50 mM/Pyr (−)] group (89% at 1 h, 77% at 3 h, 19% at 6 h, and 15% at 24 h) compared with sustained high viability ratios (> 90%) at any time point in the other groups (Fig. 1E). Cell viability was also assessed by MTS assay (Fig. 1F) and indicated more rapid (from 1 h after exposure) and extensive cell death (below the detectable level at 24 h) in the [Glc 50 mM/Pyr (−)] group compared with that seen with Trypan blue staining. The different time courses of cell death between the two assays may have been due to different measurement principles; cellular metabolic activity evaluated by MTS assay may have deteriorated quicker in the [Glc 50 mM/Pyr (−)] group compared with the cell membrane permeability evaluated by Trypan blue staining. Interestingly, pyruvate starvation-induced cell death was greater at higher glucose concentrations (5 mM < 10 mM < 25 mM < 50 mM) (Fig. 1G), and at lower pyruvate concentrations (1 mM < 0.1 mM < 0.01 and 0 mM) (Fig. 1H), but was not observed in cells cultured with 50 mM mannitol or galactose or 0.5 mM 3-deoxyglucosone (3-DG; an AGE precursor) (Fig. 1I). These findings indicated that the observed rapid Schwann cell death was attributable to the absence of exogenous pyruvate with high-glucose, but not hyperosmotic or glycative stress conditions. In addition to DMEM with 5% FBS, we prepared Tyrode’s solution with N2 supplement as basal medium that does not contain amino acids. Extensive cell death under high-glucose pyruvate starved conditions in Tyrode’s medium (Fig. 1J) can rule out the role of pyruvate anaplerosis. In addition to IMS32 Schwann cells, primary cultured adult rat DRG neurons, NSC-34 motor neuron-like cells, mouse mesangial MES13 cells, and human aortic endothelial cells (HAECs) underwent rapid and extensive cell death after exposure to 50 mM glucose in the absence of pyruvate (Fig. 2).

To explore the detailed mechanisms involved in pyruvate starvation-induced cell death under high-glucose conditions, molecular and biochemical analyses were conducted in IMS32 cells, which exhibit a high proliferative activity while retaining some characteristic features of Schwann cells and have been utilized to study diabetic neuropathy (e.g*.* polyol pathway hyperactivity, increased oxidative stress, and reduced neurotrophic factor synthesis)^11–13^.

### Pyruvate starvation under high-glucose conditions induced ROS production, but not lipid peroxidation

**Figure 3:**
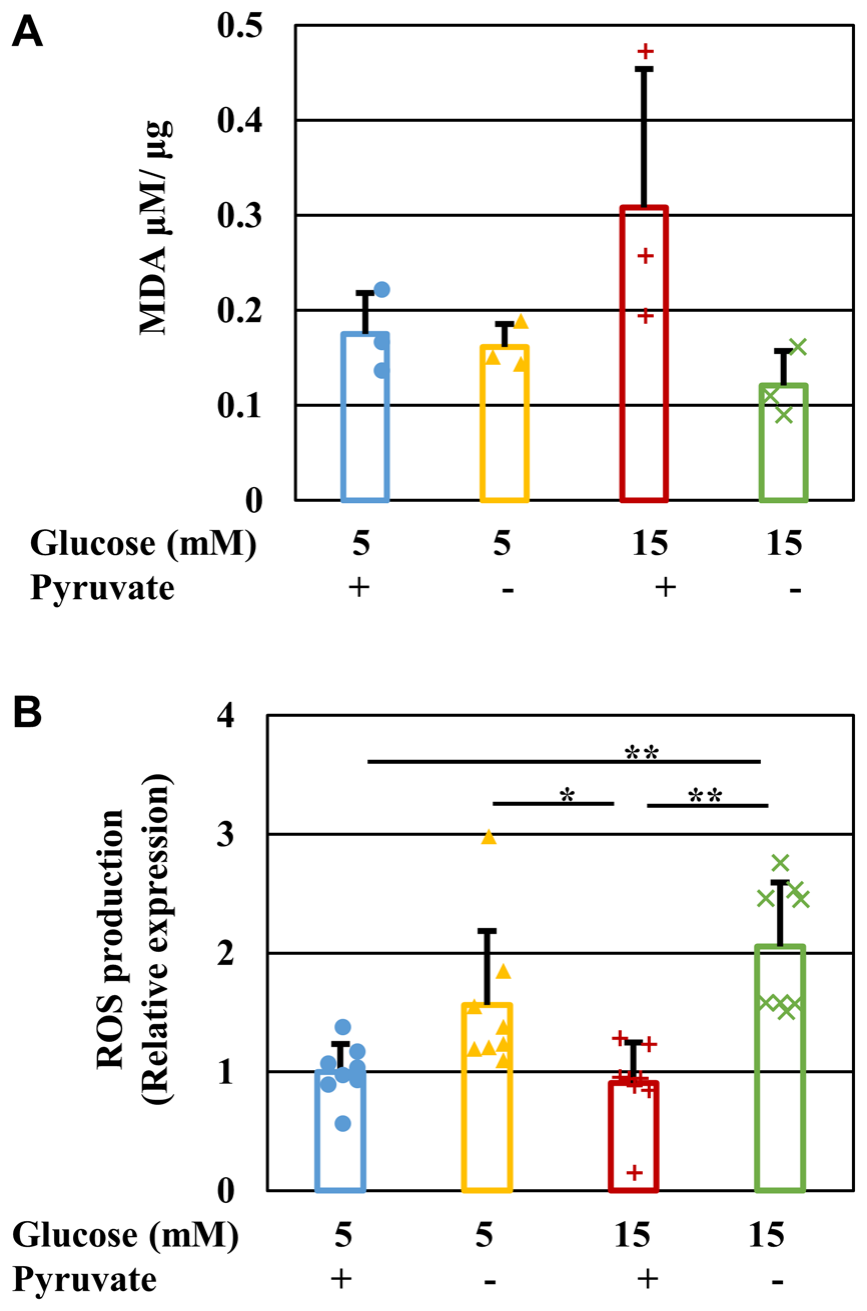
Pyruvate starvation under high-glucose conditions induces ROS production, but not lipid peroxidation. The quantity of MDA (**A**) and the fluorescent intensity of CellROX (**B**) in IMS32 cells at 1 h in the [Glc 5 mM/Pyr (+)] (blue), [Glc 5 mM/Pyr (-)] (yellow), [Glc 15 mM/Pyr (+)] (brown) and [Glc 15 mM/Pyr (-)] (green) was determined by TBARS assay (**A**) and CellROX assay (**B**), respectively. The fluorescent intensity of CellROX was normalized to ratio of the value of [Glc 5 mM/Pyr (+)]. Values represent mean + SD from three (**A**) and six (**B**) experiments (individual values are depicted as circles, triangles, pluses and crosses). ** *P* < 0.01.

Thiobarbituric Acid Reactive Substances (TBARS) and CellROX assays were used to investigate the involvement of oxidative stress in high-glucose and pyruvate starvation-induced cell death. To prevent extensive cell death during these assays, IMS32 cells were exposed to mild hyperglycemic conditions (15 mM glucose) for 1 h. There was no significant increase in the quantity of malondialdehyde (MDA), an index of lipid peroxidation, in the [Glc 15 mM/Pyr (−)] group compared with the other groups (Fig. 3A). In contrast, the fluorescent intensity of CellROX, a ROS indicator, was significantly higher in the [Glc 15 mM/Pyr (−)] group than the [Glc 15 mM/Pyr ( +)] group (Fig. 3B). These findings suggest that pyruvate starvation under high-glucose conditions induces ROS production, but not lipid peroxidation. Although no significant difference was observed, ROS production tended to be increased in the [Glc 5 mM/Pyr (−)] group compared with the [Glc 5 mM/Pyr ( +)] group.

### Pyruvate starvation under high-glucose conditions reduced mitochondrial respiration and intracellular ATP levels

**Figure 4:**
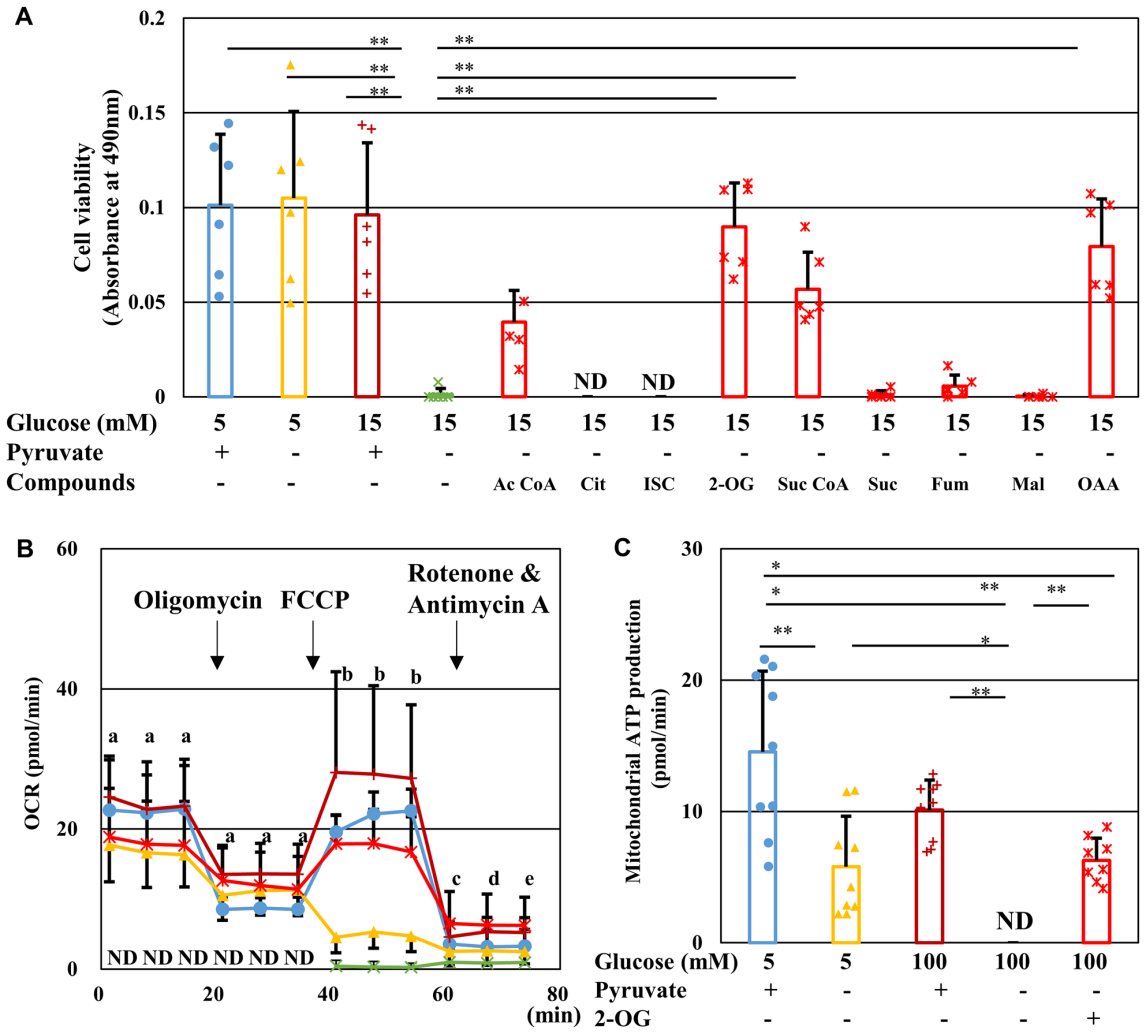
2-Oxyglutarate prevents IMS32 cell death, mitochondrial dysfunction and ATP depletion under high-glucose pyruvate-starved conditions. (**A**) Cell viability at 24 h in the [Glc 5 mM/Pyr (+)] (blue), [Glc 5 mM/Pyr (−)] (yellow), [Glc 15 mM/Pyr (+)] (brown), [Glc 15 mM/Pyr (−)] (green), and [Glc 15 mM/Pyr (−)] supplemented with TCA cycle intermediates (red) groups was determined by MTS assay. (**B**) Time course of OCR in the [Glc 5 mM/Pyr (+)] (blue), [Glc 5 mM/Pyr (−)] (yellow), [Glc 100 mM/Pyr (+)] (brown), [Glc 100 mM/Pyr (−)] (green), and [Glc 100 mM/Pyr (−)/2-OG (+)] (red) groups measured by Extracellular Flux Analyzer and Cell MitoStress Test. (**C**) Mitochondrial ATP production under these five conditions was estimated by changes in OCR through sequential addition of oligomycin and FCCP. Values represent mean + SD from six (**A**) and 9–10 (**B** and **C**) experiments (individual values are depicted as circles, triangles, pluses, crosses and asterisks). (**A** and **C**) * *P* < 0.05, ** *P* < 0.01. (**B**) [Glc 100 mM/ Pyr (−)] compared with (a) all the other groups (*P* < 0.01), (b) [Glc 5 mM/Pyr (+)], [Glc 100 mM/ Pyr (+)], or [Glc 100 mM/Pyr (−)/2-OG (+)] (*P* < 0.01), (c) [Glc 100 mM/Pyr (−)/2-OG (+)] (*P* < 0.01) or [Glc 100 mM/ Pyr (+)] (*P* < 0.05), (d) [Glc 100 mM/Pyr (−)/2-OG (+)] (*P* < 0.01), and (e) [Glc 100 mM/ Pyr (+)] or [Glc 100 mM/Pyr (−)/2-OG (+)] (*P* < 0.01). Ac CoA, acetyl CoA; Cit, citrate; ISC, isocitrate; 2-OG, 2-oxyglutarate; Suc CoA, succinyl CoA; Suc, succinate; Fum, fumarate; Mai, malate; and OAA; oxaloacetate.

The effect of pyruvate starvation on glucose metabolism in IMS32 cells was investigated using metabolomics analysis. Levels of glycolytic intermediates upstream of pyruvate, such as fructose 1,6-bisphosphate (F1,6BP) and glycelaldehyde-3-phosphate (G3P), were increased in the [Glc 15 mM/Pyr (−)] group compared with the other group. In contrast, levels of pyruvate and some TCA cycle intermediates, such as citric acid, 2-oxglutaric acid (2-OG), and fumaric acid, were diminished in the absence of exogenous pyruvate regardless of glucose concentrations (Table S1). These changes in metabolic profile were unlikely to have resulted from altered mRNA expression of the enzymes catalyzing the reactions in the glycolytic pathway and TCA cycle (Tables S2–4). In agreement with the metabolomics findings, supplementation with TCA cycle intermediates, such as acetyl CoA, 2-OG, succinyl CoA, and oxaloacetate, prevented cell death in the [Glc 15 mM/Pyr (−)] group (Fig. 4A). In a similar manner to IMS32 Schwann cells, 2-OG supplementation significantly rescued primary cultured DRG neurons from the cell death under exposure to high-glucose pyruvate-starved conditions (Fig. S1).

To verify these findings, mitochondrial respiration was evaluated in IMS32 cells using an Extracellular Flux Analyzer. Analysis of mitochondrial stress revealed that pyruvate starvation markedly decreased the baseline oxygen consumption rate (OCR) under both normal (5 mM) and high-glucose (100 mM) conditions to undetectable levels, with the most pronounced effects seen at high-glucose levels (Fig. 4B). Oligomycin was then added to inhibit ATP synthesis, carbonyl cyanide-p-trifluoromethoxyphenylhydrazone (FCCP) was added to accelerate proton intake and stimulate maximal respiration, and rotenone and antimycin A were added to inhibit mitochondrial respiratory chain function, and mitochondrial ATP production was estimated by measuring changes in OCR using sequential addition of oligomycin and FCCP (Fig. 4C). In the presence of extracellular pyruvate, the maximal OCR induced by FCCP under high-glucose conditions was significantly higher than that under normal-glucose conditions. However, in the absence of pyruvate, FCCP treatment did not upregulate OCR and remained below the detectable level under high-glucose conditions. Supplementation with 2-OG under high-glucose conditions steeply elevated OCR levels to those almost comparable to the levels observed under normal-glucose conditions in the presence of pyruvate (Fig. 4B). Moreover, 2-OG treatment prevented loss of mitochondrial ATP production induced by pyruvate starvation under high-glucose conditions (Fig. 4C). These findings suggest that cell death under high-glucose and pyruvate-deficient conditions is attributable, at least in part, to disruption of mitochondrial respiration and ATP synthesis. In the presence of pyruvate, 2-OG tended to increase OCR at the basal and oligomycin-treated phases under high-glucose conditions, but the degree of changes was mostly insignificant (Fig. S2).

### Reduced glycolytic flux and GAPDH and hexokinase activity under high-glucose pyruvate-starved conditions

**Figure 5:**
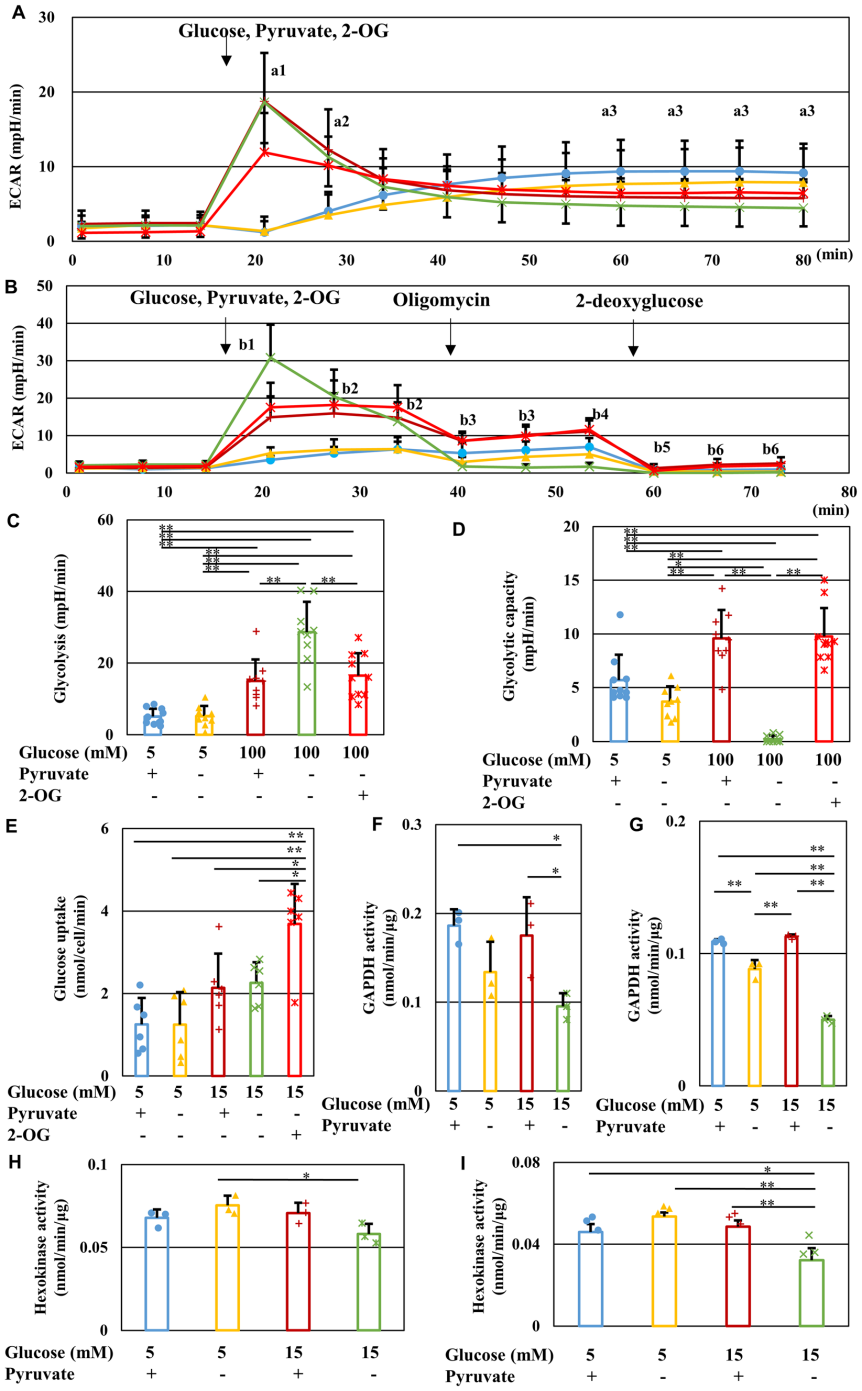
Pyruvate starvation inhibits glycolytic flux and GAPDH and hexokinase activity under high-glucose conditions. (**A**) Time course of ECAR in the [Glc 5 mM/Pyr (+)] (blue), [Glc 5 mM/Pyr (−)] (yellow), [Glc 100 mM/Pyr (+)] (brown), [Glc 100 mM/Pyr (−)] (green), and [Glc 100 mM/Pyr (−)/2-OG (+)] (red) groups measured using an Extracellular Flux Analyzer. (**B**) Time course of ECAR in the groups described as (**A**) measured using an Extracellular Flux Analyzer and XF Glycolytic test. Glucose, pyruvate and 2-OG-induced glycolysis (**C**) and maximum glycolytic capacity (**D**) under these five conditions was estimated by changes in ECAR through sequential addition of glucose, pyruvate and 2-OG, and oligomycin. (**E**) The amount of glucose uptake into IMS32 cells under the five conditions described in (**A**). GAPDH (**F, G**) and hexokinase (**H, I**) activity in IMS32 cells at 30 min (**C, E**) or 60 min (**D, F**) in the groups described in (**A**). Values represent mean + SD from 13–14 (**A**), 9–10 (**B**-**D**), six (**E**), and three (**F-I**) experiments (individual values are depicted as circles, triangles, pluses, crosses and asterisks). (**A**) [Glc 100 mM/Pyr (−)] compared with (a1) [Glc 5 mM/Pyr (+)], [Glc 5 mM/Pyr (−)], or [Glc 100 mM/Pyr (−)/2-OG (+)] (*P* < 0.01), (a2) [Glc 5 mM/Pyr (+)] or [Glc 5 mM/Pyr (−)] (*P* < 0.01), and (a3) [Glc 5 mM/Pyr (+)] (*P* < 0.01). (**B**) [Glc 100 mM/Pyr (−)] compared with (b1) [Glc 5 mM/Pyr (+)], [Glc 5 mM/Pyr (−)], [Glc 100 mM/Pyr (+)], or [Glc 100 mM/Pyr (−)/2-OG (+)] (*P* < 0.01), (b2) [Glc 5 mM/Pyr (+)], or [Glc 5 mM/Pyr (−)] (*P* < 0.01), (b3) [Glc 5 mM/Pyr (+)], [Glc 100 mM/Pyr (+)], or [Glc 100 mM/Pyr (−)/2-OG (+)] (*P* < 0.01), (b4) [Glc 5 mM/Pyr (+)], [Glc 100 mM/Pyr (+)], or [Glc 100 mM/Pyr (−)/2-OG (+)] (*P* < 0.01), and [Glc 5 mM/Pyr (−)] (*P* < 0.05), (b5) [Glc 100 mM/Pyr (+)] (*P* < 0.05), (b6) [Glc 100 mM/Pyr (+)], or [Glc 100 mM/Pyr (−)/2-OG (+)] (*P* < 0.01). (**C-I**) * *P* < 0.05, ** *P* < 0.01.

In addition to evaluating the mitochondrial respiration state, cellular glycolytic activity was examined using an Extracellular Flux Analyzer. Under normal-glucose conditions, pyruvate starvation showed no significant effect on the gradual increase in extracellular acidification rate (ECAR). However, a high-glucose load induced a rapid increase followed by a subsequent gradual decrease in ECAR, which was more enhanced in the absence of pyruvate. Supplementation with 2-OG reduced the degree of upregulation and subsequent downregulation of ECAR in high-glucose pyruvate-deficient conditions (Fig. 5A). XF Glycolysis test using an Extracellular Flux Analyzer revealed that pyruvate starvation initially increased glucose-induced glycolysis but decreased the maximum glycolytic capacity under high-glucose conditions. Supplementation with 2-OG restored these changes under those conditions (Fig. 5B-D). These results suggest that 2-OG restores transient enhancement and subsequent inhibition of glycolytic flux in IMS32 cells exposed to high-glucose pyruvate-deficient conditions. In the presence of pyruvate, supplementation with 2-OG tended to enhance ECAR under normal- and high-glucose conditions, but the degree of changes was mostly insignificant (Fig. S2). These data do not support the combined effects of 2-OG and pyruvate.

Glucose uptake in IMS32 cells tended to be enhanced by exposure to high (15 mM) glucose in the presence or absence of pyruvate, and was significantly increased by supplementation with 2-OG (Fig. 5E). Since pyruvate starvation impaired glycolysis without affecting glucose uptake under high-glucose conditions, this indicated that exogenous pyruvate plays a pivotal role in the maintenance of glycolytic flux in response to increasing intracellular glucose levels. Increased levels of F1,6BP and G3P compared with decreased levels of pyruvate and some TCA cycle intermediates (Table S1) implied there was disruption of the pathways between G3P and pyruvate in the glycolytic pathway under high-glucose pyruvate-deficient conditions. Consistent with these findings, pyruvate starvation-induced cell death under high-glucose conditions was prevented by supplementation with PEP and 3-phosphoglyceric acid (3PG), a glycolytic intermediate downstream of G3P, but not F1,6BP, which is upstream of G3P (Fig. S3). Hexokinase and G3P dehydrogenase (GAPDH) are the enzymes catalyzing the first and middle step of the glycolytic pathway, respectively. The activity of GAPDH, but not hexokinase, was reduced at 30 min after exposure to [Glc 15 mM/Pyr(-)] as compared with [Glc 15 mM/Pyr( +)], whereas both activities were significantly reduced at 60 min after exposure to that condition (Figs. 5F-I). These findings imply that pyruvate starvation induces antidromic inhibition of the glycolytic pathway under high-glucose conditions.

### Involvement of polyol pathway hyperactivity in high-glucose and pyruvate starvation-induced cell death

**Figure 6:**
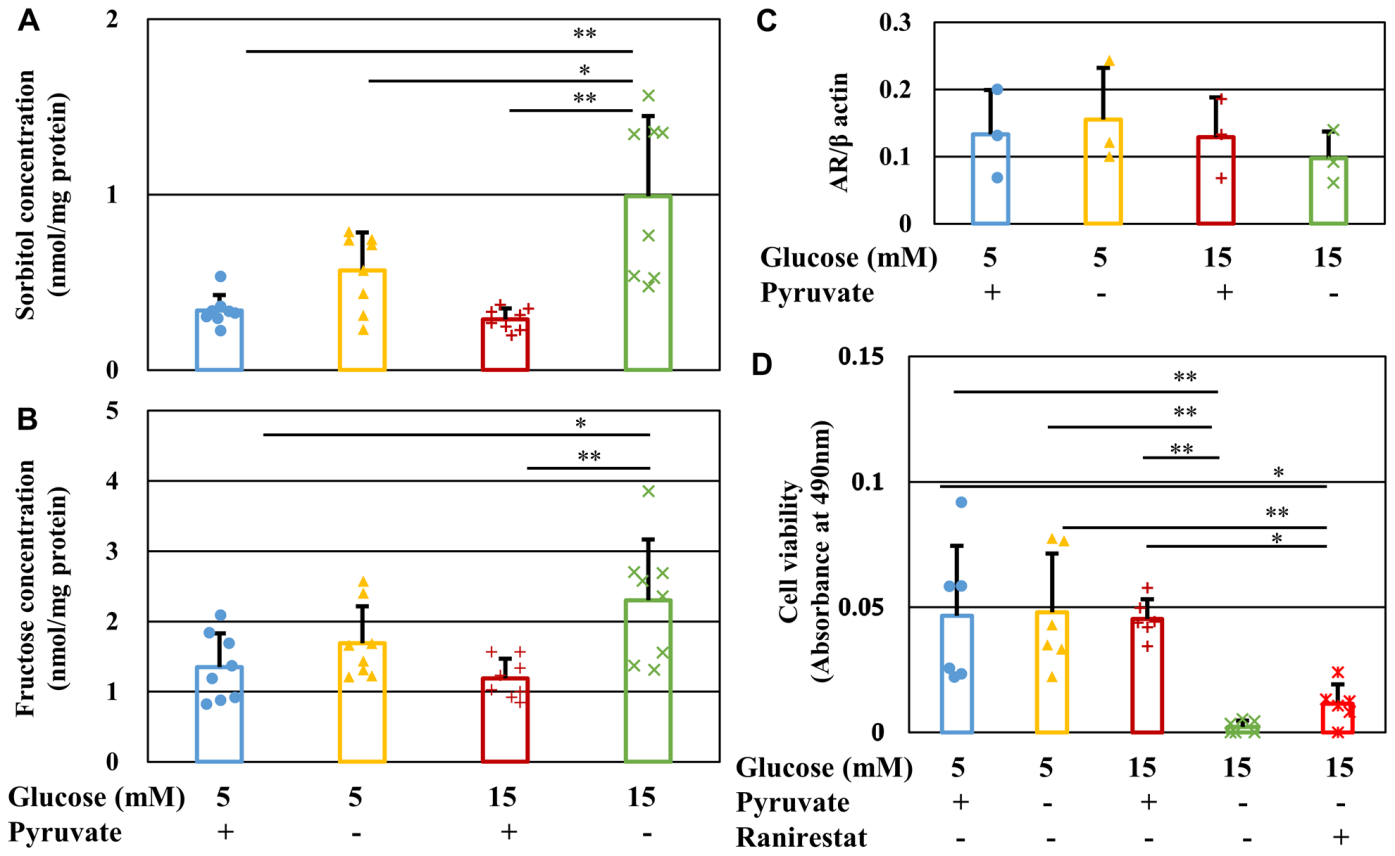
Pyruvate starvation enhances the polyol pathway under high-glucose conditions. (**A** and **B**) Concentration of sorbitol (**A**) and fructose (**B**) in IMS32 cells at 6 h in the [Glc 5 mM/Pyr (+)] (blue), [Glc 5 mM/Pyr (−)] (yellow), [Glc 15 mM/Pyr (+)] (brown), and [Glc 15 mM/Pyr (−)] (green) groups as determined by LC/MS/MS. (**C**) Relative AR protein expression in IMS32 cells at 1 h in the four groups as measured by Western blotting. Blots for AR and β-actin are shown in Fig. S4A. (**D**) The AR inhibitor, ranirestat, partially improved the viability of IMS32 cells under high-glucose pyruvate-starved conditions. Cell viability at 24 h after exposure to the four conditions described above and [Glc 15 mM/Pyr (−)/Ranirestat (+)] (**red**) was determined by MTS assay. Values represent mean + SD from eight (**A** and **B**), three (**C**), and six (**D**) experiments (individual values are depicted as circles, triangles, pluses, crosses and asterisks). * *P* < 0.05, ** *P* < 0.01.

Increased glucose uptake accompanied by reduced glycolytic flux and hexokinase activity in IMS32 cells under high-glucose pyruvate-deficient conditions implied enhanced flux into the collateral glycolysis pathways. The polyol pathway is the most upstream collateral pathway through which glucose is converted to sorbitol and fructose. Surprisingly, short-term (6 h) exposure to pyruvate starvation significantly increased sorbitol and fructose levels under both normal and high-glucose conditions ([Glc 5 mM/Pyr (−)] and [Glc 15 mM/Pyr (−)] groups), especially under high-glucose conditions, compared with the [Glc 15 mM/Pyr ( +)] group (*P* < 0.01) (Fig. 6A, B). Aldose reductase (AR) is a rate-determining enzyme in the polyol pathway that catalyzes the conversion of glucose to sorbitol. AR protein expression in IMS32 cells remained unchanged following exposure to high-glucose and pyruvate-depleted conditions; however, treatment with the AR inhibitor, ranirestat, partially prevented cell death under these conditions (Fig. 6C, D and Fig. S4A). These findings suggest that a rapid increase in flux into the polyol pathway induced by pyruvate starvation is involved in cell death under high-glucose conditions.

### Benfotiamine prevented high-glucose pyruvate-starvation-induced cell death by restoring glycolytic flux

**Figure 7:**
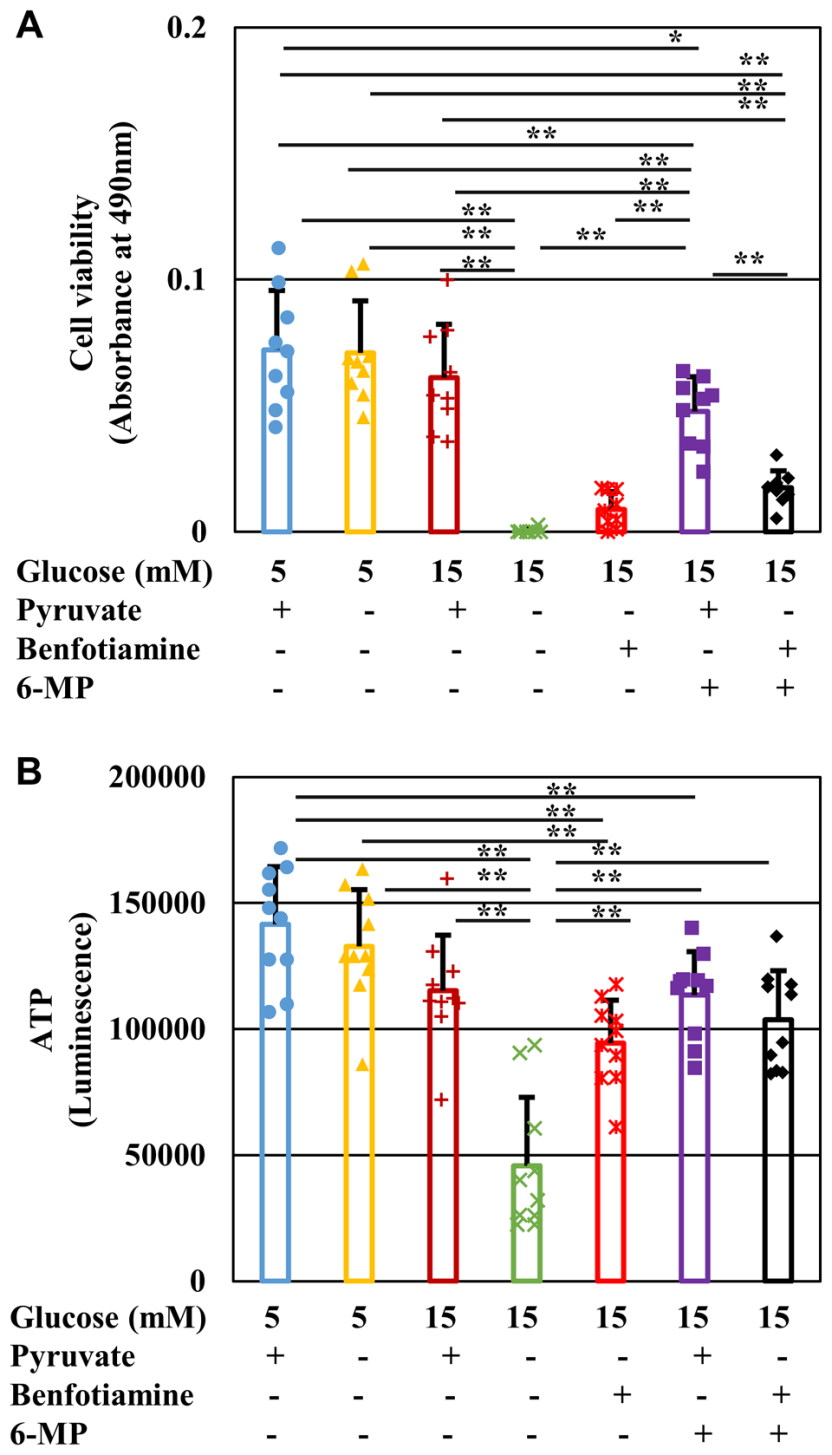
Benfotiamine prevents IMS32 cell death and ATP depletion without accelerating purine metabolism under high-glucose pyruvate-starved conditions. Cell viability (**A**) at 24 h and intracellular ATP content (**B**) at 3 h in the Glc 5 mM/Pyr (+)] (blue), [Glc 5 mM/Pyr (−)] (yellow), [Glc 15 mM/Pyr (+)] (brown), [Glc 15 mM/Pyr (−)] (green) and [Glc 15 mM/Pyr (−)/Benfotiamine (+)] (red), [Glc 15 mM/Pyr (−)/6-mercaptopurine (6-MP) (+)] (purple), and [Glc 15 mM/Pyr (−)/Benfotiamine (+)/6-MP (+)] (black) group were determined by MTS assay (**A**) and CellTiter Glo 2.0 assay (**B**), respectively. Values represent mean + SD from nine (**A**) and 10 (**B**) experiments (individual values are depicted as circles, triangles, pluses, crosses and asterisks). * *P* < 0.05, ** *P* < 0.01. 6-MP, 6-mercaptopurine.

**Figure 8:**
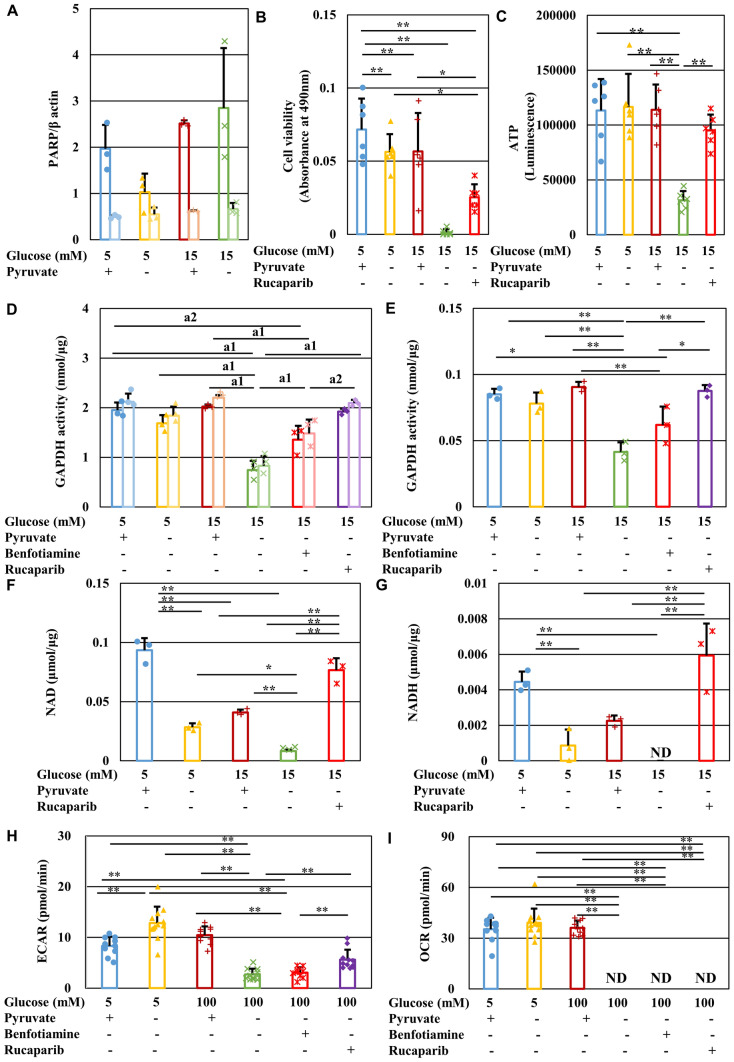
PARP is involved in IMS32 cell death under high-glucose pyruvate-starved conditions by suppressing GAPDH activity and glycolytic flux. (**A**) Relative protein expression of full-length (dark colors) and cleaved (light colors) PARP in IMS32 cells at 1 h in the [Glc 5 mM/Pyr (+)] (blue), [Glc 5 mM/Pyr (−)] (yellow), [Glc 15 mM/Pyr (+)] (brown), and [Glc 15 mM/Pyr (−)] (green) groups was determined by Western blotting. Blots for PARP and β-actin are shown in Fig. S4B. (**B** and **C**) The PARP inhibitor, rucaparib, prevented cell death and ATP depletion under high-glucose pyruvate-starved conditions. Cell viability (**B**) at 24 h and intracellular ATP contents (**C**) at 3 h in the four groups described above and the [Glc 15 mM/Pyr (−)/Rucaparib (+)] (red) group were determined by MTS assay (**B**) and CellTiter Glo 2.0 assay (**C**). (**D** and **E**) Treatment with rucaparib (purple) ameliorated the decline in basal GAPDH activity at 4 min (dark colors) and 6 min (light colors) after the initiation of measurement (**D**) and GAPDH activity/min (**E**) under high-glucose pyruvate-starved conditions. Intracellular contents of NAD (**F**) and NADH (**G**) at 1 h under these conditions described as (**B**) were determined. (**H** and **I**). Rucaparib (purple) improved ECAR (**F**) to a greater extent than benfotiamine (**red**), whereas neither agent could restore mitochondrial respiration (**G**) under high-glucose pyruvate-starved conditions. Values represent the mean + SD from three (**A, F** and **G**), eight (**B** and **C**), three (**D** and **E**), and 9–12 (**H** and **I**) experiments (individual values are depicted as circles, triangles, pluses, crosses and asterisks). (**D**) (a1) Basal GAPDH activity at 4 and 6 min (*P* < 0.01), (a2) 4 min (*P* < 0.05), and 6 min (*P* < 0.01). * *P* < 0.05, ** *P* < 0.01.

The vitamin B1 derivative, benfotiamine, suppresses flux into the collateral glycolysis pathways of hyperglycemic damage (hexosamine pathway, AGE pathway, and diacylglycerol pathway) by activating transketolase, the rate-limiting enzyme of the pentose phosphate pathway, and has been suggested as a potential therapeutic agent for diabetic complications^14^. Addition of benfotiamine to IMS32 cells under high-glucose (15 mM) pyruvate-deficient conditions prevented cell death (Fig. 7A) and depletion of intracellular ATP levels (Fig. 7B). The shift from glycolysis to the pentose phosphate pathway by benfotiamine is linked to purine synthesis, through which ATP is produced. However, 6-mercaptopurine, an inhibitor of purine nucleotide synthesis via the de novo and salvage pathways, failed to cancel the restorative effects of benfotiamine under high-glucose (15 mM) pyruvate-deficient conditions (Fig. 7). Therefore, the purine synthetic pathway is unlikely to be involved in the rescuing of cell death brought about by benfotiamine. Further investigation using an Extracellular Flux Analyzer revealed that benfotiamine tended to increase GAPDH activity and ECAR, but not OCR, under high-glucose (15 mM) pyruvate-deficient conditions (Fig. 8D, E, H, I). These findings imply that benfotiamine protects IMS32 cells from cell death via restoration of GAPDH activity and intracellular ATP levels generated by glycolytic flux.

### PARP activation under high-glucose pyruvate-starved conditions inhibited GAPDH activity and glycolysis

Poly (ADP-ribose) polymerase (PARP) is a key enzyme involved in protein modification pathways, such as poly ADP-ribosylation. Although exposure of IMS32 cells to high-glucose (15 mM) conditions in the presence or absence of pyruvate showed no significant effects on the expression of full-length and cleaved PARP (Fig. 8A and Fig. S4B), the PARP inhibitor, rucaparib, ameliorated the reduced cell viability, ATP production, GAPDH activity, and NAD and NADH levels observed under high-glucose pyruvate-starved conditions (Fig. 8B–G). In addition, rucaparib ameliorated the reduced ECAR more effectively than benfotiamine (Fig. 8H), whereas neither agent improved OCR (Fig. 8H, I). Thus, these agents likely rescued cell death under high-glucose pyruvate-starved conditions by ameliorating glycolytic flux rather than mitochondrial respiration, and the cell death observed under these conditions may be, at least in part, attributed to PARP activation that led to loss of NAD and ATP production during glycolysis.

## Discussion

We observed that the absence of exogenous pyruvate under high-glucose conditions induces time and glucose concentration-dependent cell death of immortalized mouse Schwann cells (IMS32), as well as primary cultured rat DRG neurons, lined mouse motor neurons (NSC-34), mesangial cells (MES13), and HAECs. Although several studies showed the efficacy of pyruvate supplementation in diabetes and its complications by employing experimental diabetic animals^4–6^, the critical role of exogenous pyruvate in survival and energy production in various cell types during exposure to high-glucose has not yet been documented and we believe these findings are novel and biologically significant. Glucose and pyruvate are added to most culture media as a carbon source for cultured cells, and the deleterious effects of high-glucose as well as the antioxidant actions of pyruvate have been independently discussed. Although metabolic disorders in neurons, Schwann cells and vascular endothelial cells induced by hyperglycemia are recognized as major causes of diabetic neuropathy^1^, it remains controversial whether exposure to high-glucose leads to the apparent death of these cells in vivo and in vitro^15^. In our previous studies using DRG neurons^16^ and IMS32 Schwann cells^11^, no significant differences were observed in the cell viability ratios after seven days of culture between normal-glucose (5–5.6 mM) and high-glucose (30–56 mM) conditions in the presence of pyruvate. However, in the present study, most DRG neurons and IMS32 cells died within 6 h after exposure to high (50 mM) glucose in the absence of pyruvate, whereas nearly 90% of these cells survived for 24 h under normal (5 mM) glucose conditions, regardless of the presence of pyruvate, as well as high-glucose conditions in the presence of pyruvate. Therefore, high-glucose conditions per se are unlikely to affect the viability of neurons and Schwann cells. However, it remains unclear how pyruvate starvation leads to rapid and extensive cell death under high-glucose conditions.

As previously mentioned, the antioxidant actions of pyruvate may play a role in the prevention of high-glucose-induced cell death. Exogenous pyruvate incorporated into rat cortical neurons via particular MCTs was shown to be effective in preventing H_2_O_2_-induced cell death^2^. In another study, media containing pyruvate showed lower levels of H_2_O_2_ production in Chinese hamster ovary cells since pyruvate functions as a H_2_O_2_ scavenger^17^. However, it is unreasonable to conclude that high-glucose pyruvate starvation-induced cell death is simply attributable to oxidative stress. In our study using CellROX and TBARS assays, pyruvate starvation enhanced ROS production, but not lipid peroxidation in IMS32 cells under high-glucose conditions. Since lipid peroxidation comprises a ROS-mediated chain of reactions that results in damage to membrane lipids, the discrepancy in the results between the two assays indicates that the degree of ROS production is not sufficient to cause lipid peroxidation under these conditions. Therefore, while oxidative stress may be involved in cell death, it does not appear to be a critical factor, and another role of pyruvate as ‘a promoter of glucose metabolism with energy production’ needs to be carefully considered.

Pyruvate starvation under high-glucose conditions impaired glycolytic flux and mitochondrial respiration without affecting glucose uptake, suggesting that exogenous pyruvate plays a major role in maintaining glycolysis–TCA cycle flux in response to increasing intracellular glucose levels. Glucose overload is known to saturate glycolytic flux and enhance the collateral glycolysis pathways of hyperglycemic damage (polyol pathway, hexosamine pathway, AGE pathway, and diacylglycerol pathway) in neuronal, mesangial and endothelial cells^18–20^. Therefore, pyruvate starvation may significantly accelerate the shift in flux from the glycolytic pathway to these collateral pathways, leading to rapid and extensive cell death. This hypothesis is supported by the findings that cell death could be partially prevented by the AR inhibitor, ranirestat, as well as benfotiamine, which suppresses flux into the collateral pathways. We observed that hyperglycemic insults (≥ 30 mM) in the presence of pyruvate for 7–14 days induced significant increases in the sorbitol and fructose contents in IMS32 cells^11^. In the absence of pyruvate, however, much shorter duration (6 h) of hyperglycemic insults led to the significant upregulation of sorbitol and fructose contents. To explore the mechanisms accounting for such a drastic escalation of the polyol pathway flux under high-glucose and pyruvate-starved conditions, we focused on the sequence of metabolic changes under those conditions. The findings from Extracellular Flux Analyzer indicate rapid inhibition of mitochondrial respiration and subsequent attenuation of glycolytic pathway under those conditions. In addition, the reduction of GAPDH activity prior to the hexokinase inhibition imply that pyruvate starvation induces antidromic inhibition of the glycolytic pathway under high-glucose conditions. Taking these findings together, the rapid escalation of the polyol pathway flux may result from the following sequential metabolic changes; 1) reduced TCA cycle intermediates and mitochondrial ATP production, 2) reduced GAPDH activity and inhibition of glycolytic flux, 3) augmentation of the polyol and other collateral glycolysis pathways, 4) reduced hexokinase activity and glucose utilization in the glycolytic pathway, and 5) further augmentation of the polyol pathway flux (Fig. S5).

Several studies have implied the efficacy of exogenous pyruvate for the activation and maintenance of the glycolytic pathway. For instance, pyruvate treatment upregulated the expression of genes encoding enzymes involved in glycolysis in embryonic stem cells^21^ and prevented increases in sorbitol and diacylglycerol levels in rat skin chamber granulation tissue under high-glucose conditions^22^. Kashiwagi et al. reported that pyruvate supplementation restored H_2_O_2_-induced inhibition of the key enzymes of glycolysis, such as phosphofructokinase and GAPDH, in vascular endothelial cells under high-glucose conditions^23^. In their study, exogenous pyruvate increased the intracellular pyruvate content, followed by a decrease in the cytoplasmic NADH/NAD ratio, resulting in increased GAPDH activity. In addition, pyruvate activated the pentose phosphate pathway and increased the total cellular NADPH content, resulting in enhanced glutathione-dependent H_2_O_2_ degradation. The findings from our study and the study by Kashiwagi et al.^23^ demonstrate the efficacy of pyruvate supplementation against unfavorable events caused by exposure to high-glucose; however, its significance differs considerably between the two studies. Exogenous pyruvate was shown to function as a glycolysis promoter as well as an antioxidant under the superoxidative environments set by high-glucose and H_2_O_2_ load in the study by Kashiwagi et al.^23^, whereas the present study showed that pyruvate supplementation prevented rapid cell death under high-glucose conditions alone by sustaining glycolysis–TCA cycle flux and mitochondrial respiration.

We used an Extracellular Flux Analyzer to characterize the metabolic disorders under high-glucose pyruvate-starved conditions and showed disrupted mitochondrial respiration from the beginning of exposure compared with transient enhancement and subsequent inhibition of glycolytic flux. These findings, together with reduced amounts of pyruvate and TCA cycle intermediates in the metabolomics analysis, suggest a rapid inhibition of TCA cycle flux and a subsequent gradual decrease in glycolytic flux under high-glucose pyruvate-starved conditions. Pyruvate is the end-product of the glycolytic pathway under aerobic conditions, and its conversion to acetyl CoA is the first step in the TCA cycle in mitochondria. Previous studies using isotope (^13^C or ^14^C)-labeled glucose and pyruvate revealed that both endogenous and exogenous pyruvate are metabolized into lactate or TCA cycle intermediates in cultured neurons and glial cells^24,25^. In addition, ^13^C-labeled pyruvate injected to adult mice was converted to TCA cycle intermediates in peripheral nerves^9^, brain^26^ and liver^27^. Moreover, cytosolic pyruvate imported into the mitochondrial interspace with a proton via MPC may contribute to maintenance of mitochondrial membrane potential^28^. These findings suggest that pyruvate incorporated into cells can directly and indirectly sustain TCA cycle flux and mitochondrial function.

Pyruvate is decarboxylated into acetyl CoA by pyruvate dehydrogenase, and is carboxylated into oxaloacetate or malate by pyruvate carboxylase or malic enzyme, respectively; pyruvate carboxylation is a major pathway in pyruvate anaplerosis. A previous study using the isolated heart perfused with acetoacetate, pyruvate and hydroxymalate (a malic enzyme inhibitor) indicated that pyruvate anaplerosis plays an important role in the maintenance of physiological function and the flux from malate to 2-OG in TCA cycle^29^. These findings imply the involvement of pyruvate anaplerosis in preserving the cellular function under hyperglycemic conditions. In this study, however, 2-OG improved glycolysis and mitochondrial respiration (Fig. 4B, C and Fig. 5) whereas a PARP inhibitor rucaparib restored glycolysis, but not mitochondrial respiration, under high-glucose pyruvate-starved conditions (Fig. 8H, I). Because both substances alleviated the cell death, pyruvate appears to regulate both PARP-dependent (glycolysis) and -independent (TCA cycle) cascades under high-glucose conditions. In addition, some amino acids (e.g., aspartate, alanine, and glutamate) are recognized as anaplerotic substates, as well as pyruvate^30^. IMS32 cells were maintained in Tyrode’s solution with N2 supplement, which medium does not contain these amino acids. Exposure to high-glucose pyruvate-starved conditions in Tyrode’s medium induced IMS32 cell death (Fig. 1J). These findings indicate that the cell death under high-glucose conditions can be attributed to the depletion of pyruvate, but not the amino acids. Moreover, the cell viability under high-glucose conditions depended on pyruvate concentration (Fig. 1H), which findings suggest unique roles of exogenous pyruvate in maintaining glycolysis and TCA cycle flux, beyond its role as an anaplerotic substrate under high-glucose conditions.

There is accumulating evidence showing that PARP activation is closely associated with the development of diabetic neuropathy^31,32^ and downregulates GAPDH activity via poly ADP-ribosylation of the enzyme in endothelial cells^33,34^. Moreover, overactivation of PARP1 depleted levels of NAD and ATP in astrocytes^35^. Since the PARP inhibitor, rucaparib, ameliorated the reduced viability and ECAR in IMS32 cells under high-glucose pyruvate-starved conditions, PARP activation under these conditions may suppress GAPDH activity and glycolytic flux, leading to depleted levels of NAD and ATP that may cause IMS32 cell death. In agreement with this hypothesis, the poly ADP-ribosylation-induced reduction of GAPDH activity was shown to increase flux into the collateral pathways of hyperglycemic damage in vitro^34^.

Benfotiamine is incorporated into cells and converted to the active form, thiamine diphosphate (TDP), which functions as a coenzyme for transketolase, pyruvate dehydrogenase, and α-ketoglutarate dehydrogenase. The addition of benfotiamine to endothelial cells under high-glucose conditions led to activation of transketolase, which suppressed the flux into the polyol and other collateral pathways and accelerated flux into the pentose phosphate pathway. In the present study, however, we failed to obtain the evidence that the benfotiamine-induced metabolic shift from the collateral pathways to the pentose phosphate pathway was involved in the amelioration of reduced IMS32 cell viability under high-glucose pyruvate-deficient conditions. Interestingly, benfotiamine restored ATP levels, GAPDH activity and ECAR, but not OCR, under such conditions. Glycolysis consists of the initial preparatory phase from glucose to G3P with ATP consumption and the subsequent pay-off phase from G3P to pyruvate with ATP formation, and GAPDH catalyzes the first reaction of the pay-off phase. Thus, it seems plausible that benfotiamine prevents cell death under such conditions by sustaining ATP production via the pay-off phase of glycolysis, but not mitochondrial respiration. In agreement with these findings, benfotiamine exhibited no significant effects on the activity of enzymes in TCA cycle, such as pyruvate dehydrogenase or α-ketoglutarate dehydrogenase in diabetic retinopathy^14,36^. In addition to the beneficial effects on TDP, it was reported that the thiamine metabolite, adenosine thiamine triphosphate (AThTP), inhibited PARP1 activity in vitro^37^. In obese diabetic rats, hepatic ADP-ribosylation was suppressed by thiamine administration^39^. Taken together with our results, these findings imply that AThTP converted from benfothiamine may inhibit overactivation of PARP1, which may lead to downregulation of GAPDH activity and consumption of ATP under high-glucose conditions in the absence of pyruvate.

In summary, our findings indicate that pyruvate starvation under high-glucose conditions leads to rapid IMS32 Schwann cell death via the following mechanisms: reduced mitochondrial respiration and ATP production; enhanced flux into the collateral glycolysis pathways of hyperglycemic damage; and reduced GAPDH activity and glycolytic flux, possibly due to enhanced PARP activity (Fig. S5). Therefore, exogenous pyruvate likely plays a major role in maintaining glycolysis–TCA cycle flux and energy production in cultured cells under exposure to high-glucose insult. While these findings further our understanding of cellular metabolism under diabetic conditions, our study has several limitations. First, in terms of the relevance of our study to diabetic neuropathy in vivo, it is important to note that it would be difficult to create a state of ‘absence of extracellular pyruvate under hyperglycemia’ in experimental animals. Furthermore, it remains to be elucidated whether pyruvate levels in the sera and/or peripheral nerves of humans and animals are reduced by diabetes. Second, the efficacy of exogenous pyruvate toward the PNS function under diabetic conditions should be explored in detail. Future studies are required to examine this further, with a particular focus on the restorative effects of sodium pyruvate, an investigational drug toward mitochondrial disease^39^, on animal models and patients with diabetic neuropathy.

## Materials and methods

### Materials

The compounds used in this study were galactose (Wako, Osaka, Japan), 3-deoxyglucosone (3-DG; Toronto Research Chemicals Inc., North York, Canada), citrate (Wako), fumarate, malate (Nacalai Tesque Inc., Kyoto, Japan), acetyl CoA, isocitrate, 2-oxoglutarate, succinyl CoA, succinate, oxaloacetate, phosphoenolpyruvic acid monopotassium salt, benfotiamine, fructose 1,6-bisphosphate (F1,6BP), 3-phosphoglycelic acid (3PG), mannitol (Sigma-Aldrich Co. LCC, St Louis, MO, USA), and rucaparib (Selleck Chemicals, Houston, TX, USA). The AR inhibitor, ranirestat, was provided by Sumitomo Dainippon Pharma Co., Ltd. (Osaka, Japan) as a collaborative research agreement.

### Cell culture

Spontaneously immortalized adult mouse IMS32 Schwann cells were established and passaged by our laboratory (formerly named ALS/neuropathy project)^40^. IMS32 cells were maintained in Dulbecco’s Modified Eagle’s medium (DMEM) containing 5.6 mM glucose and 1 mM sodium pyruvate (Sigma) supplemented with 5% FBS (Thermo Fisher Scientific Inc., Waltham, MA, USA) and Antibiotic–Antimycotic Mixed Solution (100 unit/mL Penicillin, 100 μg/mL Streptomycin, 250 ng/mL Amphotericin B; Nakarai Tesque). Images of the cells were observed and recorded using a phase-contrast microscope (IMT-2; Olympus, Tokyo, Japan) equipped with a microscope digital camera system (DP22-CU; Olympus) and image analysis software (WinROOF2015; Mitani Corporation, Tokyo, Japan).

Primary culture of adult rat DRG neurons was performed as previously described^41^. Three-month-old female Wistar rats were purchased from CLEA Japan, Inc. (Shizuoka, Japan) and fed standard chow and water ad libitum and were housed in a temperature-and humidity control room with 12:12 h light–dark cycle. Rats were kept in a cage (22 cm × 22 cm × 13.8 cm), and all rats received humane care and handling in accordance with the ARRIVE guidelines. All experiments were approved by the Institutional Review Board of Tokyo Metropolitan Institute of Medical Science (institutional approval number 20–007 and 20–016, 2020) and conducted in accordance with the Guidelines for the Care and Use of Animals of Tokyo Metropolitan Institute of Medical Science (2011).

NSC-34 cells produced by fusion of motor neuron-enriched embryonic mouse spinal cord cells with mouse neuroblastoma cells^42^ were gifted from Prof. Kazuhiko Watabe (Kyorin University, Tokyo, Japan), and MES13 cells established from mouse mesangial cells and HAECs were gifted from Dr. Keiichiro Matoba (The Jikei University School of Medicine, Tokyo, Japan). NSC-34 cells and MES13 cells were maintained in DMEM supplemented with 5% and 10% FBS, respectively, whereas HAEC cells were maintained in Endothelial Cell Growth Medium MV 2 Kit (PromoCell, Heidelberg, Germany).

### Cell viability assays

Glucose and pyruvate-free DMEM (Thermo Fisher Scientific, #11,966,025) supplemented with 5% FBS was used as a basal medium in the following assays. Tyrode’s solution (Sigma) with N2 supplement were also used for the assays. IMS32 cells seeded on 96-well plates at an approximate density of 9 × 10^3^ cells/cm^2^ were incubated for 1, 3, 6, or 24 h in basal medium supplemented with 5, 10, 15, 25, or 50 mM glucose (Sigma) in the presence or absence of 0.01, 0.1 or 1 mM pyruvate (Sigma). Cell viability (MTS assay) was assessed using the CellTiter 96 AQueous One Solution Cell Proliferation Assay kit (Promega, Madison, WI, USA) according to the manufacturer’s instructions.

IMS32 cells and DRG neurons seeded in 8-well chamber slides (Thermo Fisher Scientific) or 12-well plates were maintained in the same culture conditions as above, and dead cells were detected using positive Trypan blue staining. Cells in each well were incubated with Trypan blue solution (Nakarai Tesuque) for 15 min at 37 °C, and the number of viable (Trypan blue-negative) cells was counted under a phase-contrast microscope.

NSC-34, MES13, and HAEC cells were seeded on 96-well plates and incubated for 24 h in medium containing 5 or 50 mM glucose in the presence or absence of 1 mM pyruvate, and the viability under each culture condition was assessed in a similar manner used for IMS32 cells.

### Measurement of ROS production

IMS32 cells were seeded at a density of 9 × 10^3^ cells/cm^2^ and incubated for 1 h in DMEM/5%FBS containing 5 or 15 mM glucose in the presence or absence of 1 mM pyruvate including 5 μM CellROX green reagent (Thermo Fisher Scientific). intracellular ROS production was assessed under each culture condition at a wavelength of 485/520 nm (excitation/emission) using a plate reader.

### Measurement of lipid peroxidation

IMS32 cells were seeded at a density of 9 × 10^3^ cells/cm^2^ and maintained for 1 h under each condition described as *Measurement of ROS production*. Intracellular MDA levels were assessed under each condition by TBARS assay (Cayman Chemical Company, Ann Arbor, MI, USA) and a plate reader according to the manufacturer’s instructions.

### Measurement of sorbitol and fructose levels in IMS32 cells using liquid chromatography coupled with tandem mass spectrometry (LC/MS/MS)

IMS32 cells were seeded at a density of 9 × 10^3^ cells/cm^2^ and incubated for 6 h under each condition described as *Measurement of ROS production*. Intracellular concentrations of sorbitol and fructose were determined using the LC/MS/MS system at Sumika Chemical Analysis Service, Ltd. (Osaka, Japan) as part of a collaborative research agreement with Sumitomo Dainippon Pharma Co., Ltd. (Osaka, Japan)^44^.

### Microarray analysis

IMS32 cells were seeded at a density of 9 × 10^3^ cells/cm^2^ and incubated for 1 h under each condition described as *Measurement of ROS production*. Total RNA was extracted from the cells using TRIzol solution (Thermo Fisher Scientific) and the quality of the RNA was assessed by electrophoresis and a bioanalyzer (Agilent Technologies, Santa Clara, CA, USA). Cy3-labelled cRNA was prepared using a Low Input Quick Amp Labeling Kit (Agilent Technologies) according to the manufacturer’s instructions. The labelled samples were hybridized to Agilent Whole Mouse Genome version 2.0 Microarray (G4846A), washed, and scanned using a SureScan Microarray Scanner (Agilent Technologies). Microarray data were measured using Feature Extraction software (Agilent Technologies) and analyzed using GeneSpring GX software (Agilent Technologies). The GEO accession number for microarray data reported in this paper is GSE156433.

### Metabolomics analysis

IMS32 cells were seeded at a density of 9 × 10^3^ cells/cm^2^ and maintained for 1 h under each condition described as *Measurement of ROS production*. Metabolomic analysis was performed, according to the manufacturer’s instructions (C-Scope, Human Metabolome Technologies, Inc., Tsuruoka, Yamagata, Japan). Briefly, cells were rinsed in 5% mannitol solution and immersed in methanol containing internal standards (Human Metabolome Technologies, Inc). After ultrafiltration at 9,100 × *g* at 4 °C for 35 min using a filter (Ultrafree-MC PLHCC, Human Metabolome Technologies, Inc), the samples were stored at − 80 °C until analyzed. The quantitative analysis of metabolites was conducted by Human Metabolome Technologies, Inc using Agilent capillary electrophoresis time-of-flight mass spectrometry system Machine No. 6 (cation measurement) and Agilent CE system and Agilent 6460 TripleQuad LC/MS Machine No. QqQ1 (anion measurement). The systems were connected by a fused silica capillary (50 μm i.d. × 80 cm total length) with electrophoresis buffer (H3301-1001 and H3302-1021 for cation and anion analyses, respectively, Human Metabolome Technologies, Inc.) as the electrolyte (C-SCOPE, Human Metabolome Technologies, Inc). Agilent capillary electrophoresis time-of-flight mass spectrometry and Agilent CE system and Agilent 6460 TripleQuad LC/MS peaks were automatically extracted by MasterHands (Keio university) and MassHunter Quantitative Analysis B.06.00 (Agilent Technologies), respectively, in order to obtain peak information, including *m/z*, peak area, and migration time. Peaks were annotated according to the Human Metabolome Technologies, Inc metabolite library based on *m/z* and migration time. Levels of metabolites were normalized to the internal standards and sample area and were calculated according to a calibration curve.

### Western blotting

IMS32 cells were seeded at a density of 9 × 10^3^ cells/cm^2^ and maintained for 1 h under each condition described as *Measurement of ROS production*. Western blot analysis was performed as previously described^44^ with slight modifications. Briefly, cells were dissolved in RIPA buffer (Wako) supplemented with protease inhibitor cocktails (Takara Bio Inc, Shiga, Japan). The cell lysate was sonicated using a handy sonic (TOMY SEIKO Co., Ltd., Tokyo, Japan). Protein electrophoresis and transfer were conducted using NuPAGE system (Thermo Fisher). Target proteins were visualized using the following antibodies: mouse anti-β-actin monoclonal antibody (1:2000; Sigma-Aldrich)^43^; goat anti-AR polyclonal antibody (Santa Cruz Biotechnology, Inc., Santa Cruz, TX, USA)^43^; rabbit anti-PARP polyclonal antibody (1:1000; Cell Signaling Technology, Beverly, MA, USA); horseradish peroxidase-conjugated anti-mouse IgG, anti-rabbit IgG and anti-goat IgG (1:2000, MBL Corp., Ltd., Nagoya, Japan). Bands were visualized using ECL plus Western blotting detection kit (GE Healthcare). The signal intensity was quantified using chemiluminescence imaging system EZ capture II (ATTO, Tokyo, Japan), and the relative intensity of each protein was expressed as the intensity of each protein divided by the intensity of β-actin.

### Extracellular Flux Analyzer measurements

OCR and ECAR were assessed using XFe96 Extracellular Flux Analyzer (Agilent Technologies) according to the manufacturer’s manual. IMS32 cells were seeded in 96-well assay plates (Agilent Technologies) at a density of 1 × 10^4^ cells and allowed to adhere overnight. For OCR measurement, cells were incubated for 1 h at 37 °C in DMEM (Agilent Technologies) containing 5 or 100 mM glucose in the presence or absence of 1 mM pyruvate. OCR analyses were performed using XF Cell MitoStress Test (Agilent Technologies) by sequentially injecting oligomycin, FCCP, and rotenone/antimycin A. The final concentrations of oligomycin, FCCP, and rotenone/antimycin A used in the experiments were 1, 2 and 0.5 μM, respectively. For ECAR measurement, the cells were incubated for 1 h at 37 °C in glucose and pyruvate-free DMEM. At approximately 20 min after initiation of measurement, glucose and pyruvate were injected into the wells and the measurements were continued for 40 min. Glycolytic flux was also evaluated using XF Glycolysis test (Agilent Technologies) by sequentially injecting glucose, pyruvate and 2-OG, oligomycin and 2-deoxyglucose.

### Measurement of ATP levels

IMS32 cells were seeded at a density of 9 × 10^3^ cells/cm^2^ and maintained for 3 h under each condition described as *Measurement of ROS production*. Intracellular ATP levels under each condition were assessed using the CellTiter Glo 2.0 assay kit (Promega) according to the manufacturer’s instructions.

### Measurement of GAPDH activity, hexokinase activity, glucose uptake, and NAD and NADH levels

IMS32 cells were seeded at a density of 9 × 10^3^ cells/cm^2^ and maintained for 30 or 60 min under each condition described as *Measurement of ROS production*. GAPDH activity, hexokinase activity, glucose uptake, and NAD and NADH levels under each culture condition were quantified using Glyceraldehyde 3 Phosphate Dehydrogenase assay kit (AbCam), Hexokinase Colorimetric Assay Kit (Sigma-Aldrich), Glucose Uptake-Glo Assay kit (Promega) and NAD/NADH Assay Kit-WST (DOJINDO Laboratories, Kumamoto, Japan), respectively, according to the manufacturer’s instructions.

### Statistical analysis

All the data are presented as mean + standard deviation (SD). All statistical analyses were performed using Easy R (EZR)^44^. Values below the detection limit are expressed as ND (not detected). Statistical analysis of all data was performed by one-way analysis of variance (ANOVA) followed by post hoc comparisons with Tukey HSD test, or Mann–Whitney test. All *P*-values < 0.05 between the groups were considered statistically significant.

## Supplementary Information

Supplementary Information.

## Data Availability

All data presented in this paper are available in the manuscript or from the corresponding author (H. Y).
