# Supplementary material for: Role of pyruvate in maintaining cell viability and energy production under high-glucose conditions
Source: Sci Rep. 2021 Sep 23;11:18910. doi: 10.1038/s41598-021-98082-w (PMC8460646; doi:10.1038/s41598-021-98082-w)
Supplement: Supplementary file 1 — Supplementary Information. [file 41598_2021_98082_MOESM1_ESM.pdf]

Supplementary material

Role of pyruvate in maintaining cell viability and energy production under high-glucose conditions

Hideji Yako, Naoko Niimi, Ayako Kato, Shizuka Takaku, Yasuaki Tatsumi, Yasumasa Nishito, Koichi Kato, Kazunori Sango

Supporting Information: Tables (4) and Figures (5)

Table S1

[Table S1] Metabolome analysis; the levels of metabolites involved in glycolysis, TCA cycle, pentose phosphate pathway and purine synthetic pathway in IMS32 cells at 1 h in the [Glc 5 mM/Pyr (+)], [Glc 5 mM/Pyr (-)], [Glc 15 mM/Pyr (+)] and [Glc 15 mM/Pyr (-)].

ND: not detected.

Compound	Concentration (pmol/10^6 cells)			
	[Glc 5 mM/Pyr (+)]	[Glc 5 mM/Pyr (-)]	[Glc 15 mM/Pyr (+)]	[Glc 15 mM/Pyr (-)]
Glucose 6-phosphate	53	41	48	46
Fructose 6-phosphate	19	ND	3.6	25
Fructose 1,6-bisphosphate	1,015	583	1,457	2,119
Glyceraldehyde 3-phosphate	ND	19	ND	260
Dihydroxyacetone phosphate	ND	109	47	795
3-Phosphoglyceric acid	54	62	68	71
2-Phosphoglyceric acid	2.1	3.4	3.1	3.0
Phosphoenolpyruvate	ND	ND	ND	ND
Pyruvic acid	1,191	ND	3,151	560
Lactatic acid	10,962	14,594	26,997	18,898
Acetyl CoA	16	18	4.0	6.4
Citric acid	3,481	2,594	3,696	2,277
cis-Aconitic acid	35	9.7	24	2.0
Isocitric acid	ND	ND	ND	ND
2-Oxoglutaric acid	873	249	1,020	243
Succinic acid	656	608	666	552
Fumaric acid	349	ND	529	ND
Malic acid	2,545	545	4,163	724
6-Phosphogluconate	6.6	ND	ND	ND
Ribulose 5-phosphate	0.2	13	2.1	28
Ribose 5-phosphate	5.0	5.6	4.4	36
Xylulose 5-phosphate	ND	ND	ND	ND
Sedoheptulose 7-phosphate	ND	ND	ND	ND
Erythrose 4-phosphate	ND	161	ND	ND
Ribose 1-phosphate	ND	ND	ND	ND
ADP-ribose	34	35	22	23
PRPP	9.5	16	16	11
IMP	51	36	36	78
Inosine	ND	ND	ND	ND
XMP	2.9	3.3	ND	1.8
Adenylosuccinic acid	9.2	8.5	13	9.9
AMP	523	518	441	294
ADP	1,841	2,013	2,320	1,650
ATP	10,636	11,361	12,873	10,514
NAD^+	1,729	1,839	1,936	1,212
NADH	110	134	183	175
$NADP^+$	144	126	130	103
NADPH	82	87	121	108

Table S2

[Table S2] DNA microarray analysis; the relative expression of genes encoding glycolytic enzymes in IMS32 cells at 1 h in the [Glc 5 mM/Pyr (+)], [Glc 5 mM/Pyr (-)], [Glc 15 mM/Pyr (+)] and [Glc 15 mM/Pyr (-)].

ND: not detected.

Gene	Protein	5 mM glucose pyruvate+	5 mM glucose pyruvate-	15 mM glucose pyruvate+	15 mM glucose pyruvate-	15 mM glucose pyruvate- /15 mM glucose pyruvate+
Hk1	Hexokinase 1	55.72	53.28	56.08	52.79	0.94
Hk2	Hexokinase 2	1.08	1.05	1.06	0.95	0.90
Hk3	Hexokinase 3	0.01	0.02	0.01	0.01	0.80
G6pd2	Glucose-6-phosphate dehydrogenase 2	0.10	0.09	0.10	0.12	1.22
Pfkfb1	6-phosphofructo-2-kinase/fructose-2,6-bisphosphatase 1	0.59	0.58	0.58	0.45	0.77
Pfkfb2	6-phosphofructo-2-kinase/fructose-2,6-bisphosphatase 2	0.15	0.14	0.13	0.13	0.97
Pfkfb3	6-phosphofructo-2-kinase/fructose-2,6-bisphosphatase 3	0.98	1.09	0.94	1.11	1.18
Pfkfb4	6-phosphofructo-2-kinase/fructose-2,6-bisphosphatase 4	1.87	1.97	2.11	1.91	0.91
Aldoa	Aldolase A	96.94	97.41	106.91	112.99	1.06
Aldob	Aldolase B	ND	ND	ND	ND	ND
Aldoc	Aldolase C	0.38	0.40	0.42	0.58	1.38
Tpi1	Triosephosphate Isomerase 1	29.91	31.75	32.05	34.35	1.07
Gapdh	Glyceraldehyde 3-phosphate dehydrogenase	102.74	110.52	109.72	106.62	0.97
Pgk1	Phosphoglycerate kinase 1	43.40	42.99	42.56	46.64	1.10
Pgk2	Phosphoglycerate kinase 2	0.02	0.02	0.02	0.02	1.04
Pgam1	Phosphoglycerate mutase 1	57.52	58.11	59.24	60.45	1.02
Pgam2	Phosphoglycerate mutase 2	0.05	0.04	0.04	0.05	1.04
Pgam5	Phosphoglycerate mutase 5	8.77	8.89	9.06	10.53	1.16
Eno1	Enolase 1	61.53	62.80	62.73	62.85	1.00
Eno1b	Enolase 1b	58.90	59.31	62.26	60.43	0.97
Eno2	Enolase 2	1.66	1.70	1.49	1.70	1.14
Eno3	Enolase 3	3.05	3.34	3.31	3.63	1.10
Eno4	Enolase 4	0.03	0.02	0.03	0.03	1.04
Pklr	Pyruvate kinase isotype L/R	0.03	0.02	0.02	0.02	1.03
Pkm	Pyruvate kinase	31.59	32.83	34.37	34.67	1.01

Table S3

[Table S3] DNA microarray analysis; the relative expression of genes encoding enzymes of collateral glucose-utilising pathways in IMS32 cells at 1 h in the [Glc 5 mM/Pyr (+)], [Glc 5 mM/Pyr (-)], [Glc 15 mM/Pyr (+)] and [Glc 15 mM/Pyr (-)].

Gene	Protein	5 mM glucose pyruvate+	5 mM glucose pyruvate-	15 mM glucose pyruvate+	15 mM glucose pyruvate-	15 mM glucose pyruvate-/15 mM glucose pyruvate+
Tkt	Transketolase	32.49	33.64	34.86	32.96	0.95
Gfpt1	Glutamine-fructose-6-phosphate aminotransferase 1	2.91	2.80	2.73	2.48	0.91
Gfpt2	Glutamine-fructose-6-phosphate aminotransferase 2	0.06	0.07	0.07	0.08	1.09
Akr1b3	Aldose reductase	24.42	24.82	25.73	27.06	1.05
Sord	Sorbitol dehydrogenase	4.84	5.25	5.28	5.77	1.09

Table S4

[Table S4] DNA microarray analysis; the relative expression of genes encoding enzymes of TCA cycle in IMS32 cells at 1 h in the [Glc 5 mM/Pyr (+)], [Glc 5 mM/Pyr (-)], [Glc 15 mM/Pyr (+)] and [Glc 15 mM/Pyr (-)].

Gene	Protein	5 mM glucose pyruvate+	5 mM glucose pyruvate-	15 mM glucose pyruvate+	15 mM glucose pyruvate-	15 mM glucose pyruvate-/15 mM glucose pyruvate+
Cs	Citrate synthase	54.22	54.28	56.97	53.79	0.94
Aco1	Aconitase 1	0.07	0.08	0.06	0.07	1.08
Aco2	Aconitase 2	12.19	12.30	12.85	12.19	0.95
Idh1	Isocitrate dehydrogenase (NADP(+)) 1	0.33	0.38	0.37	0.42	1.14
Idh2	Isocitrate dehydrogenase (NADP(+)) 2	29.15	29.20	31.37	31.34	1.00
Idh3a	Isocitrate Dehydrogenase (NAD(+)) 3 Catalytic Subunit Alpha	9.32	8.60	8.81	9.63	1.09
Idh3b	Isocitrate Dehydrogenase (NAD(+)) 3 Non-Catalytic Subunit Beta	4.35	4.29	4.53	4.74	1.05
Idh3g	Isocitrate Dehydrogenase (NAD(+)) 3 Non-Catalytic Subunit Gamma	19.99	19.00	19.79	22.13	1.12
Ogdh	Oxoglutarate dehydrogenase	9.65	10.01	9.72	8.85	0.91
Sucla2	Succinate-CoA Ligase ADP-Forming Subunit Beta	4.34	4.17	4.40	4.72	1.07
Suclg1	Succinate-CoA Ligase GDP/ADP-Forming Subunit Alpha	6.49	6.28	6.77	7.18	1.06
Suclg2	Succinate-CoA Ligase GDP-Forming Subunit Beta	0.73	0.63	0.68	0.67	0.99
Sdha	Succinate Dehydrogenase Complex Flavoprotein Subunit A	9.12	8.99	9.35	9.48	1.01
Sdhb	Succinate Dehydrogenase Complex Iron Sulfur Subunit B	33.92	34.23	37.53	38.31	1.02
Sdhc	Succinate Dehydrogenase Complex Subunit C	9.48	9.58	9.84	10.61	1.08
Sdhd	Succinate Dehydrogenase Complex Subunit D	17.21	16.31	18.79	20.84	1.11
Fh1	Fumarate hydratase 1	12.58	12.35	13.68	13.47	0.98
Mdh1	Malate Dehydrogenase 1	17.24	17.58	19.12	19.77	1.03
Mdh1b	Malate Dehydrogenase 1B	0.13	0.14	0.15	0.16	1.05
Mdh2	Malate Dehydrogenase 2	77.00	81.56	86.56	87.10	1.01

Fig. S1

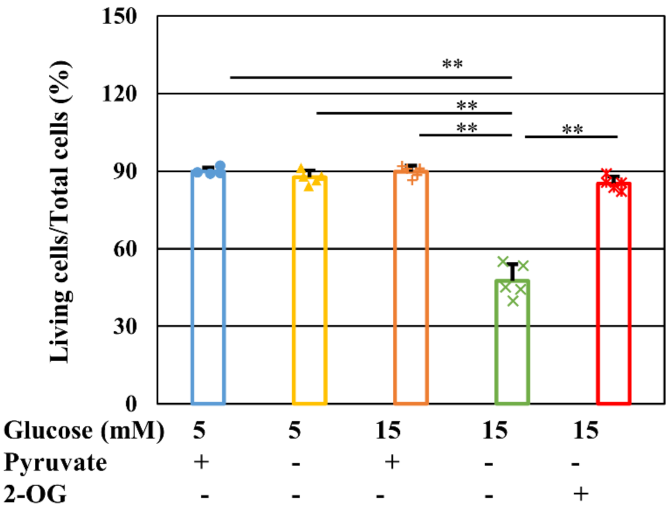

[Figure S1] 2-OG prevents DRG neuronal death under high-glucose pyruvate-starved conditions

Cell viability at 6 h in the [Glc 5 mM/Pyr (+)] (**blue**), [Glc 5 mM/Pyr (−)] (**yellow**), [Glc 15 mM/Pyr (+)] (**brown**), [Glc 15 mM/Pyr (−)] (**green**), and [Glc 15 mM/Pyr (−)/2-OG (+)] (**red**) groups was determined by trypan blue staining. Data represent mean + SD from 4-5 values from independent culture (individual values are depicted as circles, triangles, pluses, crosses and asterisks). Statistical analysis of the data was performed by one-way ANOVA followed by post hoc comparisons with Tukey HSD test. ** P<0.01.

Fig. S2

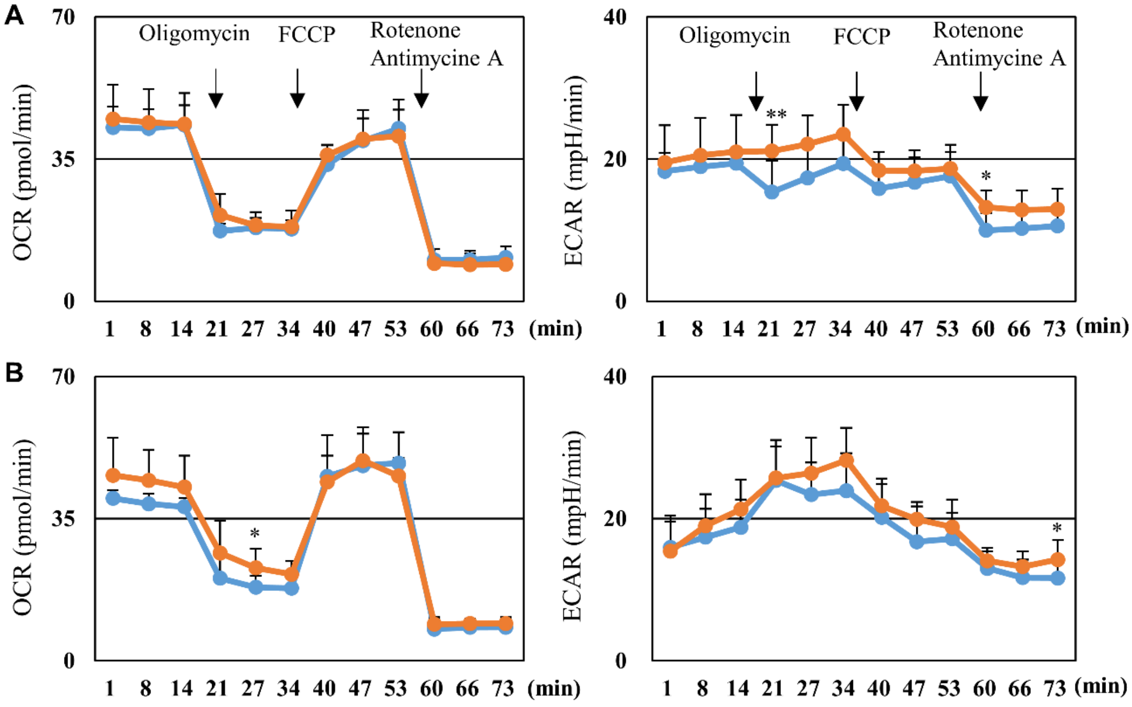

[Figure S2] The effects of 2-OG supplementation on OCR and ECAR under normal- and high-glucose conditions in the presence of pyruvate

Time courses of OCR (**left**) and ECAR (**right**) in the [Glc 5 mM/Pyr (+)/2-OG(-)] (**A**, blue), [Glc 5 mM/Pyr(+)/2-OG(+)] (**A**, orange), [Glc 100 mM/Pyr (+)/2-OG(-)] (**B**, blue), and [Glc 100 mM/Pyr (+)/2-OG(+)](**B**, orange) groups measured by Extracellular Flux Analyzer. Data represent mean + SD from 9-10 values from two independent cultures. Statistical analysis of the data was performed Mann-Whitney test. * $P<0.05$, ** $P<0.01$.

Fig. S3

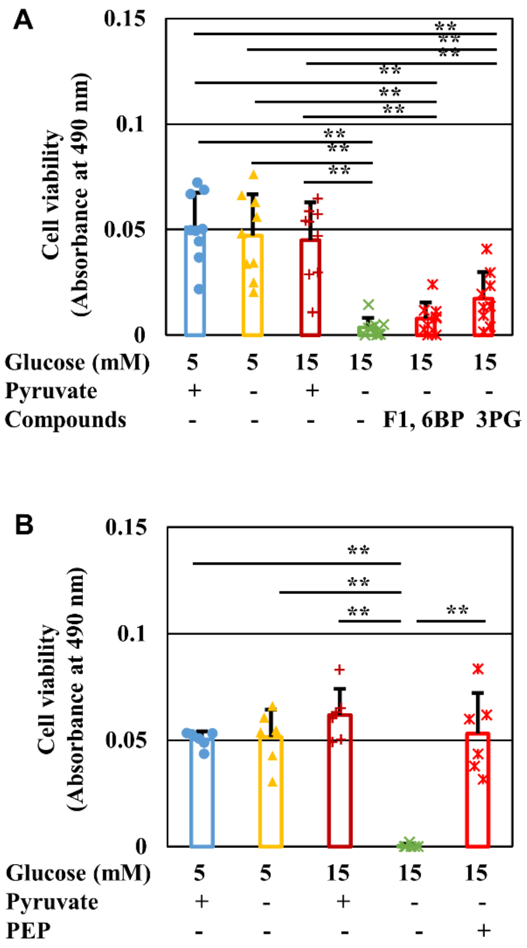

[Figure S3] 3PG and PEP, but not F1,6BP, prevents IMS32 cell death under high-glucose pyruvate-starved conditions

Cell viability at 24 h in the [Glc 5 mM/Pyr (+)] (**blue**), [Glc 5 mM/Pyr (−)] (**yellow**), [Glc 15 mM/Pyr (+)] (**brown**), [Glc 15 mM/Pyr (−)] (**green**), and [Glc 15 mM/Pyr (−)] supplemented with (**A**) F1,6BP, 3-phosphogryceric acid (3-PG) or (**B**) phosphoenolpyruvate (PEP) (**red**) groups was determined by MTS assay. Data represent mean + SD from six (**A**) and nine (**B**) values from two or three independent cultures (individual values are depicted as circles, triangles, pluses, crosses and asterisks). ** *P*<0.01.

Fig. S4

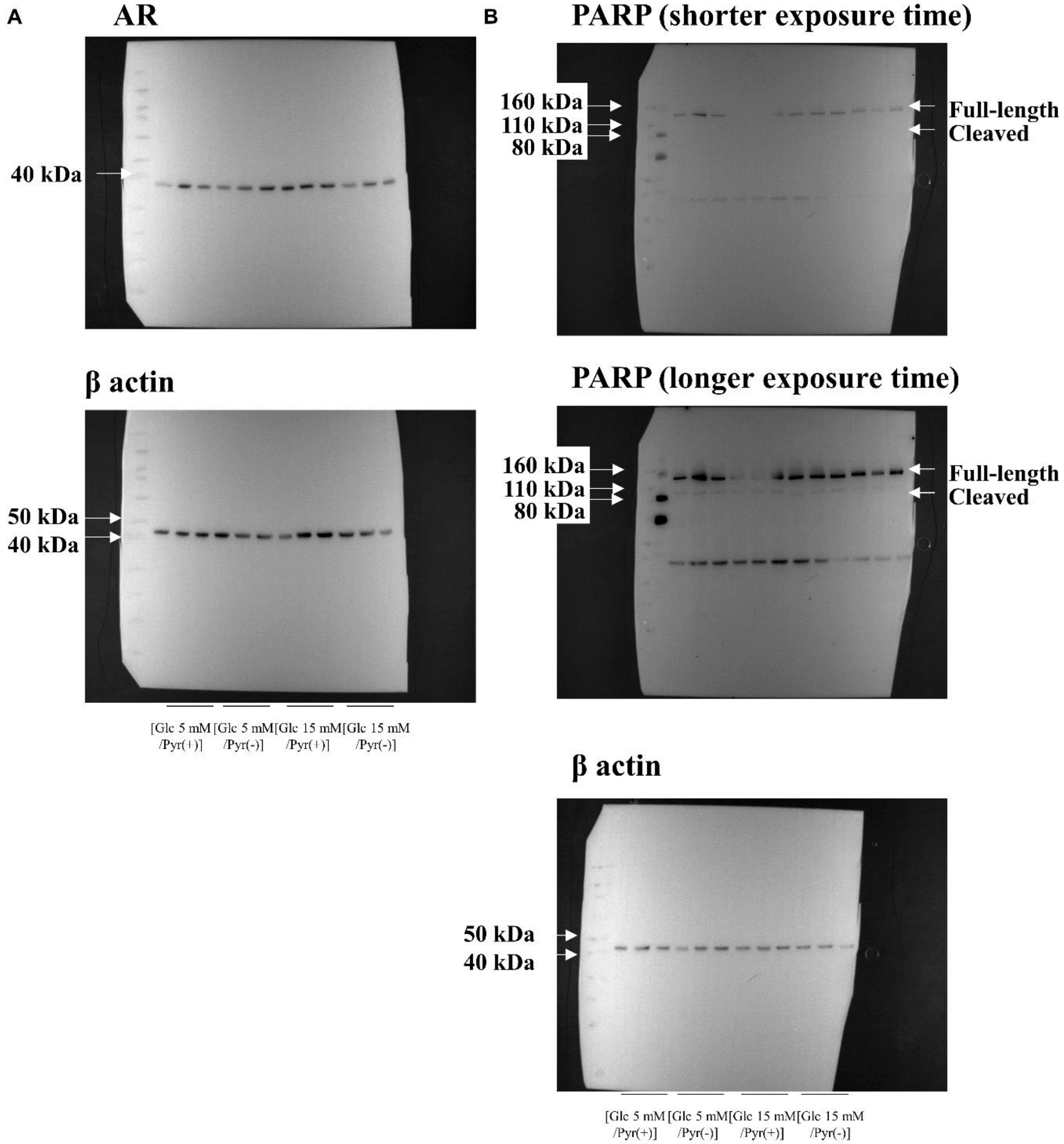

[Figure S4] Actual Western blot pictures of AR and β actin (A), and PARP (full-length and cleaved) and β actin (B).

Full length of the membranes presented in Fig. 6C (A) and Fig. 8A (B). AR and β actin (A), and PARP in shorter and longer exposure time and β actin (B) were sequentially visualized in same membranes.

Fig. S5

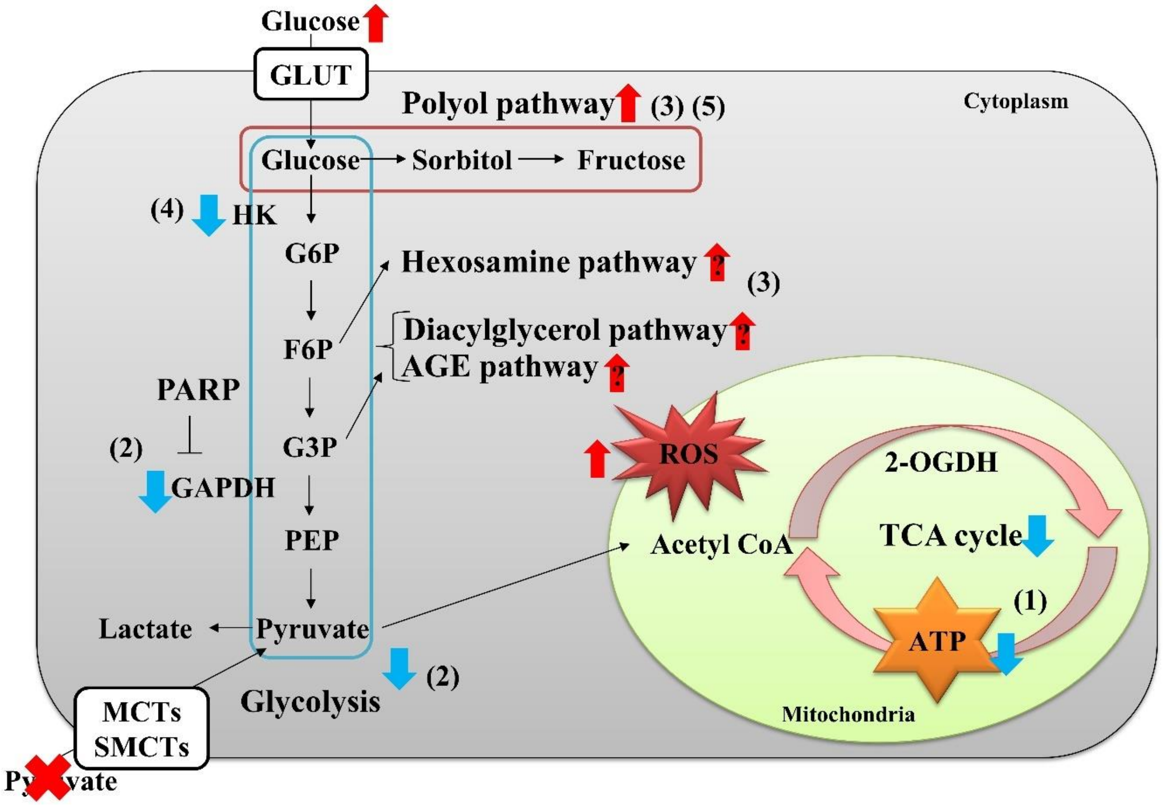

[Figure S5] The schematic representation of sequential metabolic change in IMS32 cells under high-glucose pyruvate-starved conditions.

Rapid IMS32 cell death under high-glucose pyruvate-starved conditions can be caused by the sequential metabolic changes as follows; 1) reduced TCA cycle intermediates and mitochondrial ATP production, 2) impaired GAPDH activity and inhibition of glycolytic flux, 3) augmentation of the polyol and other collateral glycolysis pathways, 4) decreased hexokinase (HK) activity and glucose utilization in the glycolytic pathway, and 5) further enhancement of the polyol pathway flux. The schematic image was created by H. Y.
